# Chronological Changes in MicroRNA Expression in the Developing Human Brain

**DOI:** 10.1371/journal.pone.0060480

**Published:** 2013-04-16

**Authors:** Michael P. Moreau, Shannon E. Bruse, Rebecka Jornsten, Yushi Liu, Linda M. Brzustowicz

**Affiliations:** 1 Department of Genetics, Rutgers University, Piscataway, New Jersey, United States of America; 2 Lovelace Respiratory Research Institute, Albuquerque, New Mexico, United States of America; 3 Mathematical Sciences, University of Gothenburg and Chalmers University of Technology, Gothenburg, Sweden; French National Center for Scientific Research – Institut de biologie moléculaire et cellulaire, France

## Abstract

**Objective:**

MicroRNAs (miRNAs) are endogenously expressed noncoding RNA molecules that are believed to regulate multiple neurobiological processes. Expression studies have revealed distinct temporal expression patterns in the developing rodent and porcine brain, but comprehensive profiling in the developing human brain has not been previously reported.

**Methods:**

We performed microarray and TaqMan-based expression analysis of all annotated mature miRNAs (miRBase 10.0) as well as 373 novel, predicted miRNAs. Expression levels were measured in 48 post-mortem brain tissue samples, representing gestational ages 14–24 weeks, as well as early postnatal and adult time points.

**Results:**

Expression levels of 312 miRNAs changed significantly between at least two of the broad age categories, defined as fetal, young, and adult.

**Conclusions:**

We have constructed a miRNA expression atlas of the developing human brain, and we propose a classification scheme to guide future studies of neurobiological function.

## Introduction

MicroRNAs (miRNAs) form a growing class of endogenous non-coding RNA molecules that modulate gene expression post-transcriptionally. Like transcription factors, an increase in the number of miRNAs strongly correlates with greater organismal complexity [1]. miRNAs form elaborate and sophisticated regulatory networks, where a given miRNA can influence the stability or translatability of hundreds of mRNA targets [2], and numerous miRNAs can act in concert to repress a common target. While transcription factors act as “switches” to initiate broad developmental transitions, miRNAs may act downstream to fine-tune genetic regulatory programs. An influential model posits that highly expressed miRNAs act to quell the deleterious effects of leaky transcription, while moderately expressed miRNAs buffer fluctuations in expression at weak or suboptimal promoters [3].

Generation of cellular diversity during mammalian brain development requires precise coordination of gene regulatory networks, with integral involvement of miRNAs. Lineage-specific expression signatures of cultured astrocytes and neurons [4] implicate miRNAs in neural cell fate specification. Neurobiological functions have been attributed to specific miRNAs. For example, miR-124 promotes neuronal differentiation [5], and miR-134 is involved in dendritic branching [6]. Global ablation of miRNAs is achieved in model systems by knocking out the essential miRNA processing enzyme, Dicer. In zebrafish, maternal zygotic Dicer knockout embryos undergo axis formation and body patterning, but exhibit profound defects in brain morphogenesis [7]. Likewise, selective inactivation of Dicer produces morphogenetic CNS abnormalities in conditional knockout mice, including microcephaly and reduced elaboration of dendritic branches [8]. While neuronal progenitors remain viable in the absence of miRNAs, cytoarchitectural abnormalities arise from failures to differentiate and propagate newborn neurons [9]. Dysregulation of miRNAs has been implicated in the etiology of schizophrenia [10], [11], [12], [13], as well as age-related neurodegenerative disorders [14], [15], [16].

Early expression studies uncovered a subset of brain-specific or brain-enriched miRNAs [17]. Molecular profiling studies in rodents showed that miRNAs are dynamically regulated during brain development [18], with a “chronological wave” of sequentially expressed miRNA classes [19]. Since these landmark studies, hundreds of additional miRNAs have been discovered, including many that are specific to humans. In the present study, we have performed comprehensive miRNA expression analysis in human postmortem brain samples representing fetal, early postnatal, and adult time points. We measured the expression of all known miRNAs (miRBase 10.0), as well as 373 novel, putative miRNAs. Classifying miRNAs based on temporal expression profiles may provide insight into their regulation and potential neurobiological functions.

## Methods

### Sample selection and preparation

Frozen brain tissue samples were obtained from the NICHD Brain and Tissue Bank for Developmental Disorders, housed at the University of Maryland. Brain sectioning (described at http://medschool.umaryland.edu/btbank/NICHD-BTB-ProtocolMethods.asp) began with separation of the cerebral hemispheres, followed by removal of the midbrain/pons/cerebellum. The remaining cerebrum (from the left hemisphere) was sectioned coronally at approximately 1 cm intervals. A total of 48 frozen cerebral tissue samples were obtained. Fetal samples originated from subjects ranging in gestational age from 14 weeks to 24 weeks. Additional tissue samples were obtained to represent early postnatal time points. The majority of tissue samples originated from African American individuals (**Table 1**). Three samples were excluded from microarray expression analysis, two due to poor RNA integrity measures and one due to uncertain sample identification. All remaining samples had RNA integrity measures >7. The adult time point is represented by two commercially available total RNA products. FirstChoice Human Brain Total RNA (Ambion) is a high-quality RNA sample derived from an 81 year old adult male, and FirstChoice Human Brain Reference RNA (Ambion) is pooled from twenty-three male and female Caucasian donors with an average age of 68.

**Table 1:** Demographic information of brain tissue donors.

UMB#	GA weeks	Years	Days	Pool	Sex	Race	PMI (hrs)
4912	14			1	female	African AmerincaAmerican	1
4799	14			1	male	African American	3
4794	14			1	male	African American	1
4934	16			2	male	African American	2
4573	16			2	female	African American	1
1388	16			2	male	African American	1
396	16			2	male	African American	1
249	17			3	male	African American	1
12	17			3	male	African American	1
43	17			3	female	African American	1
57	18			4	female	African American	1
42	18			4	female	African American	1
33	18			4	female	African American	1
31	18			4	male	African American	1
14	18			4	female	African American	3
4825	18			4	male	African American	1
4823	18			4	male	African American	4
113	18			4	female	African American	1
201	18			4	male	African American	1
278	18			4	male	African American	1
279	18			4	female	African American	1
1521	18			4	female	African American	1
280	18			4	female	African American	2
291	18			4	male	African American	12
391	18			4	male	Caucasian	1
392	19			5	male	African American	1
4609	19			5	female	African American	2
261	19			5	male	African American	1
250	19			5	male	African American	1
66	19			5	female	African American	3
9	19			5	female	African American	2
46	19			5	female	Caucasian	12
4889	19			5	male	African American	2
4715	20			6	male	African American	4
4654	20			6	unknown	African American	2
45	20			6	male	African American	2
*311	24				female	Caucasian	1
779		0	5	7	male	African American	5
*633		0	60		male	African American	21
1055		0	96	7	male	Caucasian	12
*1742		0	89		female	African American	24
1296		0	98	7	male	African American	16
1453		1	78	8	male	African American	19
1063		1	123	8	male	African American	21
1488		1	137	8	male	African American	21
1499		4	170	9	female	Asian	21
4670		4	273	9	male	Caucasian	17
1185		4	258	9	male	Caucasian	17

samples excluded from expression analysis.

UMB# – Sample identifier, NICHD Brain and Tissues Bank for Developmental Disorders.

GA – gestational age.

Pool – indicates sample pooling for TaqMan arrays.

PMI – post-mortem interval (hours).

Tissue homogenization and RNA extraction procedures were performed using the *mir*Vana PARIS kit (Ambion) according to the manufacturer's protocol. RNA yield and quality were determined using a NanoDrop ND-1000 spectrophotometer (Thermo Fisher Scientific, Waltham, MA) and Agilent 2100 Bioanalyzer (Agilent Technologies, Santa Clara, CA).

### Microarray preparation, data acquisition, and analysis

Dual-channel microarray expression analysis was performed using the NCode Human miRNA Microarray V3 (Invitrogen, Carlsbad, CA), which contains 710 probes for validated human miRNAs from miRBase release 10.0 as well as 373 Invitrogen novel human miRNAs. On each array, a single Cy3 labeled total RNA sample was competitively hybridized against a Cy5 labeled control pool. The control pool was a mixture of all RNA samples, where each developmental time point was equally represented, and each individual sample was equally represented within a given time point. For each channel, 1 μg of total RNA was labeled with the *Label* IT® miRNA Labeling Kit, Version 2 (Mirus Bio, Madison, WI) according to manufacturer's protocol. Samples were hybridized to the array at 37°C overnight using the hybridization buffer included with the labeling kit.

Image analysis was performed on a GenePix 4000B microarray scanner. A marked difference in pixel intensity was observed between Sanger miRNAs and the novel features exclusively present on the NCode V3 arrays, with many of the latter approaching saturation. Photomultiplier tube (PMT) settings were adjusted so as to balance signal intensity in the red and green channels, and maximize pixel intensity without saturation at 10% power. Arrays were then rescanned at 100% power without PMT adjustment. The 10% and 100% scan data were subsequently merged using methods outlined in **Methods S1 in File S1**, which resulted in substantial improvements in data quantity and quality. Images were exported and processed using the Acuity 4.0 software package. Microarray data has been deposited in the Gene Expression Omnibus (GEO) database under accession number GSE45126. Data filtering parameters were established to include only features with (1) at least 70% of pixels greater than 2 standard deviations above background in either the red or green channel, (2) regression R^2^ greater than 0.5 to ensure feature uniformity, (3) less than 20% of pixels saturated in both the red and the green channels, and (4) no flags upon visual inspection. Normalization was performed with the LOWESS (locally weighted regression) method, and expression log ratios were used for statistical analysis. Merging and filtering resulted in a final set of 464 genes. Prior to significance testing, distribution assumption checks were performed, as outlined in **Methods S2 in File S1**.

464 genes which passed the filtering criteria were analyzed using ANOVA (analysis of variance) and backward model selection, and genes whose expression differed between any subset of the fetal, young, and adult time points were identified (method described in more detail in **Methods S3 in File S1**). A simulation based false discovery rate (FDR) estimation similar to the SAM procedure [20] was used to adjust for multiple testing. 2500 simulated datasets were used, and it was found that a p-value of 0.001901 controled for the FDR at a rate of 1%.

### Preparation of TaqMan miRNA arrays

Sample pools for each unique developmental age were constructed by combining equal amounts of extracted total RNA (samples used in pools indicated in **Table 1**). Reverse transcription reactions were performed using 600ng total RNA input and reagents from the TaqMan miRNA Reverse Transcription Kit and Megaplex Primer Pools (Applied Biosystems). Reactions were performed (without preamplification) according to the Megaplex Pools protocol (Applied Biosystems, Carlsbad, CA), and thermal cycling was performed in a PTC-200 DNA Engine (MJ Research). Real-time PCR reactions were performed using the TaqMan Human miRNA Array set v2.0 in a 7900HT real-time PCR system (Applied Biosystems, Carlsbad, CA). A single array set was run for each sample pool.

### Analysis of TaqMan miRNA arrays

Relative expression analysis was performed using the SDS 2.3 software. Using the 2^−ΔΔCt^ method [21], expression levels of all miRNAs were normalized to the geometric mean of RNU44 and RNU48 snoRNAs and mammalian U6 snRNA. A stringent threshold was set to exclude miRNAs which did not show robust expression in at least one time point. This threshold was arbitrary, and corresponded to a raw Ct of >29 at all time points. Next, the fold change in expression was calculated relative to the 14 week time point in order to identify miRNAs with differential expression within the prenatal time points. Similar analysis was performed, using the 5–98 day time point as a reference, for the postnatal time points. Additionally, the 14 week time point was used as a reference to calculate fold-changes at all prenatal and postnatal time points. The distribution of these fold changes was used to identify miRNAs that exceeded two standard deviation fold change at any given time point. Only mature, guide-strand derived, miRNAs were included in these analyses.

## Results

**Figure 1:**
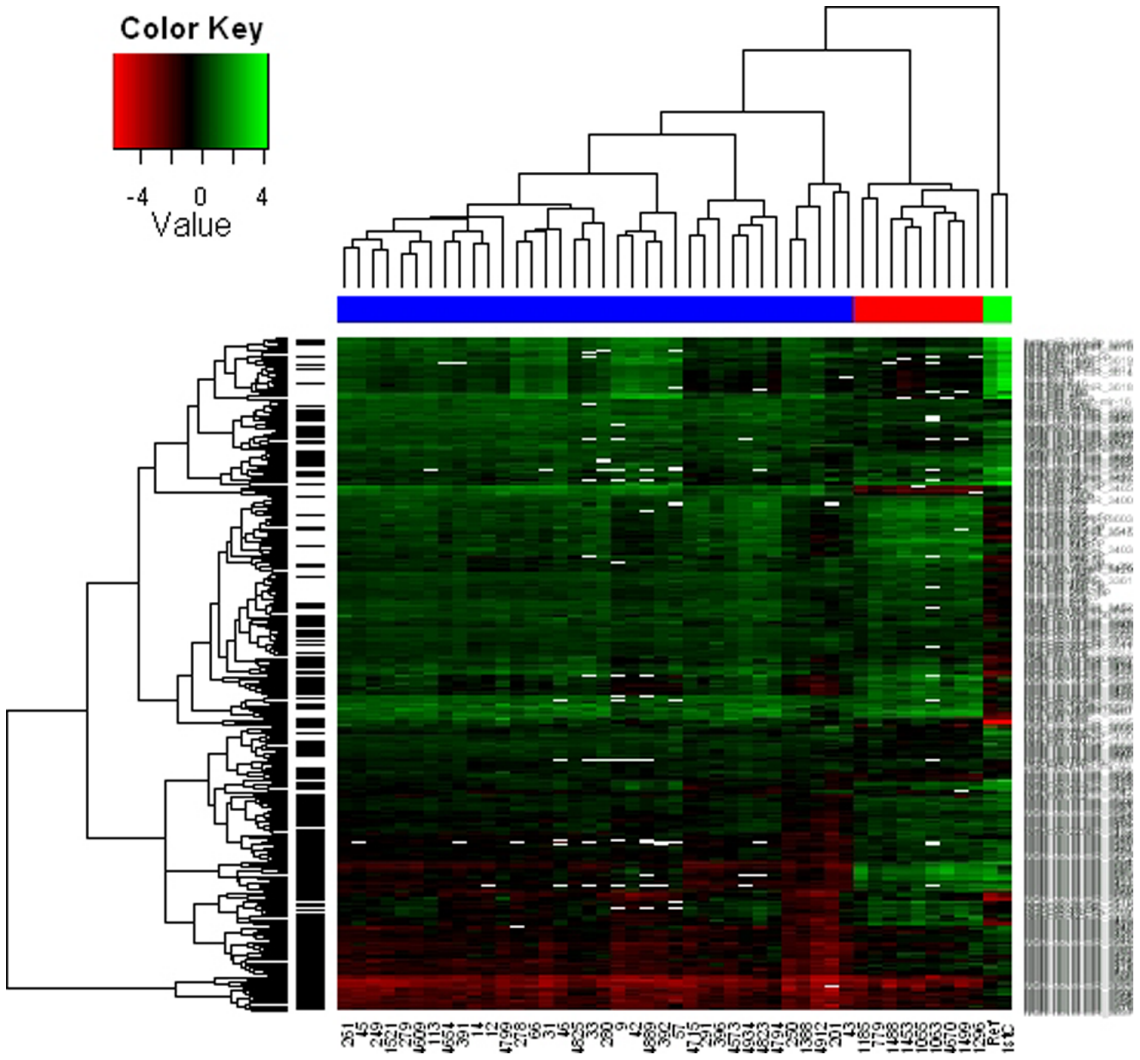
Hierarchical clustering of all significant miRNAs. Hierarchical clustering was performed on 312 differentially expressed miRNAs using a correlation metric. Included miRNAs have significantly different expression levels in at least one pair-wise comparison between the general sample types fetal, young and adult. The sample types are indicated in the colored bar (blue-fetal, red-young, green-adult).

MicroRNA expression analysis was performed using the NCode Human miRNA microarray (Invitrogen), which contains 710 probes for validated human miRNAs from miRBase release 10.0, as well as 373 Invitrogen novel human miRNAs. After filtering, the final data set consisted of 464 genes which could be assessed for differential expression between the time points. 312 had significantly different expression between at least two of the sample types, broadly defined as fetal, young and adult. Developmental expression patterns for these 312 miRNAs were analyzed using correlation-based hierarchical clustering (**Figure 1**). Samples from developmentally proximal time points had more similar miRNA expression signatures, suggesting that a global miRNA expression profile can act as a marker of developmental stage. Of the 312 significant genes; (a) 81 genes correspond to the case where F≠Y = A, i.e., the fetal mean expression is significantly different from young and adult; (b) 101 genes correspond to the case where F = Y≠A; (c) 60 genes correspond to the case where F = A≠Y; and (d) 70 genes correspond to the case where all mean expression levels differ (F≠Y≠A).

Model classes were refined to take into account the direction of expression differences between sample types, resulting in 13 unique model classes (**Table 2**). Model classes 1 and 2 are models for which the young and adult mean expression levels coincide, and the fetal mean expression differs. A substantially greater number of miRNAs belong to the class where fetal expression levels are lower than that of young and adult samples (60 miRNAs versus 14 that show higher relative expression). Model classes 1 and 7, marked by lowest expression at fetal time points, appear to be particularly enriched for novel miRNAs. Re-sampling via permutation of age category labels confirmed that this enrichment for novel miRNAs is significant (p = 0.0015 for class 1, p = 0.0393 for class 7). For model classes where the fetal and young mean expression levels coincide and adult expression differs, there is a more balanced split between down-regulation and up-regulation (model classes 3 and 4 with 41 and 60 genes, respectively). The same is also true when fetal and adult expression levels coincide, with the young being different (model classes 5 and 6 with 33 and 27 genes, respectively). Subsequent experiments investigated distinct RNA fractions to gain insight into the Invitrogen novel content (**Methods** **S4 and Figure S1 in File S1**), and those features suspected of being larger than 200 bp are indicated in **Table 2**.

**Table 2:** Classifying miRNAs based on developmental expression profiles.

Class 1: F<Y = A	Class 2: F>Y = A	Class 3: F = Y<A	Class 4: F = Y>A	Class 5: F = A<Y
hsa-miR-138	hsa-miR-17	hsa-let-7e	hsa-miR-125a-3p	hsa-miR-187*
hsa-miR-382	hsa-miR-185	hsa-let-7f	hsa-miR-142-5p	hsa-miR-220b
IVGN-novel-miR_3301	hsa-miR-516b	hsa-miR-103	hsa-miR-150*	hsa-miR-30c-1*
IVGN-novel-miR_3311	hsa-miR-654-5p	hsa-miR-107	hsa-miR-193b*	hsa-miR-425
IVGN-novel-miR_3313	IVGN-novel-miR_3408	hsa-miR-125a-5p	hsa-miR-198	hsa-miR-491-5p
IVGN-novel-miR_3314	IVGN-novel-miR_3456	hsa-miR-181c	hsa-miR-214	hsa-miR-612
IVGN-novel-miR_3318	IVGN-novel-miR_3493	hsa-miR-24	hsa-miR-23a*	hsa-miR-617
IVGN-novel-miR_3321	IVGN-novel-miR_3558	hsa-miR-26a	hsa-miR-296-3p	hsa-miR-628-3p
IVGN-novel-miR_3326	IVGN-novel-miR_3644	hsa-miR-29a	hsa-miR-298	hsa-miR-665
IVGN-novel-miR_3327	IVGN-novel-miR_3646	hsa-miR-30d	hsa-miR-30b*	hsa-miR-744
IVGN-novel-miR_3346 X	IVGN-novel-miR_3652	hsa-miR-373	hsa-miR-370	hsa-miR-92b*
IVGN-novel-miR_3351	IVGN-novel-miR_3654	hsa-miR-491-3p	hsa-miR-373*	IVGN-novel-miR_3309
IVGN-novel-miR_3356	IVGN-novel-miR_3683	hsa-miR-497	hsa-miR-422a	IVGN-novel-miR_3315
IVGN-novel-miR_3383		hsa-miR-646	hsa-miR-485-5p	IVGN-novel-miR_3385
IVGN-novel-miR_3384		hsa-miR-768-3p	hsa-miR-486-3p	IVGN-novel-miR_3386 X
IVGN-novel-miR_3387 X		hsa-miR-98	hsa-miR-518c*	IVGN-novel-miR_3429
IVGN-novel-miR_3390 X		IVGN-novel-miR_3306 X	hsa-miR-548c-5p	IVGN-novel-miR_3433
IVGN-novel-miR_3394 X		IVGN-novel-miR_3310 X	hsa-miR-564	IVGN-novel-miR_3435
IVGN-novel-miR_3397		IVGN-novel-miR_3316 X	hsa-miR-608	IVGN-novel-miR_3436
IVGN-novel-miR_3399 X		IVGN-novel-miR_3322 X	hsa-miR-610	IVGN-novel-miR_3437
IVGN-novel-miR_3401		IVGN-novel-miR_3332	hsa-miR-616	IVGN-novel-miR_3453
IVGN-novel-miR_3402		IVGN-novel-miR_3341	hsa-miR-630	IVGN-novel-miR_3463
IVGN-novel-miR_3403		IVGN-novel-miR_3354 X	hsa-miR-650	IVGN-novel-miR_3468
IVGN-novel-miR_3410 X		IVGN-novel-miR_3355	hsa-miR-659	IVGN-novel-miR_3474
IVGN-novel-miR_3411		IVGN-novel-miR_3391 X	hsa-miR-671-5p	IVGN-novel-miR_3515
IVGN-novel-miR_3412 X		IVGN-novel-miR_3422	hsa-miR-674	IVGN-novel-miR_3537
IVGN-novel-miR_3415		IVGN-novel-miR_3428	hsa-miR-760	IVGN-novel-miR_3538
IVGN-novel-miR_3418		IVGN-novel-miR_3460	hsa-miR-933	IVGN-novel-miR_3559
IVGN-novel-miR_3423		IVGN-novel-miR_3502	IVGN-novel-miR_3328	IVGN-novel-miR_3579
IVGN-novel-miR_3424		IVGN-novel-miR_3504	IVGN-novel-miR_3329	IVGN-novel-miR_3593
IVGN-novel-miR_3438 X		IVGN-novel-miR_3525	IVGN-novel-miR_3359	IVGN-novel-miR_3694
IVGN-novel-miR_3458		IVGN-novel-miR_3541	IVGN-novel-miR_3361	IVGN-novel-miR_3695
IVGN-novel-miR_3461		IVGN-novel-miR_3560	IVGN-novel-miR_3377	
IVGN-novel-miR_3465		IVGN-novel-miR_3570	IVGN-novel-miR_3378	
IVGN-novel-miR_3485		IVGN-novel-miR_3573 X	IVGN-novel-miR_3400	
IVGN-novel-miR_3486		IVGN-novel-miR_3614	IVGN-novel-miR_3425	
IVGN-novel-miR_3495		IVGN-novel-miR_3624	IVGN-novel-miR_3434	
IVGN-novel-miR_3497		IVGN-novel-miR_3628	IVGN-novel-miR_3452	
IVGN-novel-miR_3503		IVGN-novel-miR_3650	IVGN-novel-miR_3466	
IVGN-novel-miR_3520		IVGN-novel-miR_3672 X	IVGN-novel-miR_3467	
IVGN-novel-miR_3535 X		IVGN-novel-miR_3673	IVGN-novel-miR_3470	
IVGN-novel-miR_3543			IVGN-novel-miR_3471	
IVGN-novel-miR_3548			IVGN-novel-miR_3507	
IVGN-novel-miR_3550 X			IVGN-novel-miR_3516	
IVGN-novel-miR_3552			IVGN-novel-miR_3522	
IVGN-novel-miR_3553 X			IVGN-novel-miR_3526	
IVGN-novel-miR_3556			IVGN-novel-miR_3527	
IVGN-novel-miR_3557			IVGN-novel-miR_3536	
IVGN-novel-miR_3563			IVGN-novel-miR_3549	
IVGN-novel-miR_3564			IVGN-novel-miR_3586	
IVGN-novel-miR_3566 X			IVGN-novel-miR_3591	
IVGN-novel-miR_3568 X			IVGN-novel-miR_3604	
IVGN-novel-miR_3582			IVGN-novel-miR_3606	
IVGN-novel-miR_3583			IVGN-novel-miR_3649	
IVGN-novel-miR_3595			IVGN-novel-miR_3651	
IVGN-novel-miR_3599			IVGN-novel-miR_3668	
IVGN-novel-miR_3607 X			IVGN-novel-miR_3688	
IVGN-novel-miR_3626				
IVGN-novel-miR_3657				
IVGN-novel-miR_3659				
IVGN-novel-miR_3679				
IVGN-novel-miR_3680				
IVGN-novel-miR_3681				
IVGN-novel-miR_3682 X				
IVGN-novel-miR_3697 X				
IVGN-novel-miR_3698 X				
IVGN-novel-miR_3699 X				

Class 6: F = A>Y	Class 7: F<Y<A	Class 9: Y>F>A	Class 10: F>Y>A	Class 12: Y<F<A
hsa-miR-106a	hsa-miR-22	hsa-miR-129-5p	hsa-miR-505*	hsa-let-7a
hsa-miR-106b	IVGN-novel-miR_3353	hsa-miR-185*	hsa-miR-550	hsa-let-7b
hsa-miR-124	IVGN-novel-miR_3357	hsa-miR-193a-5p	hsa-miR-551b*	hsa-let-7c
hsa-miR-125b	IVGN-novel-miR_3413	hsa-miR-194*	hsa-miR-920	hsa-let-7d
hsa-miR-181a	IVGN-novel-miR_3454	hsa-miR-202	IVGN-novel-miR_3335	hsa-let-7g
hsa-miR-181b	IVGN-novel-miR_3489	hsa-miR-215		hsa-let-7i
hsa-miR-206	IVGN-novel-miR_3542	hsa-miR-25*		hsa-miR-10b
hsa-miR-20a	IVGN-novel-miR_3551	hsa-miR-30c-2*		hsa-miR-16
hsa-miR-28-5p	IVGN-novel-miR_3580	hsa-miR-371-5p		hsa-miR-195
hsa-miR-297	IVGN-novel-miR_3666	hsa-miR-449b		hsa-miR-330-3p
hsa-miR-541*		hsa-miR-498		hsa-miR-574-5p X
hsa-miR-9		hsa-miR-557		hsa-miR-768-5p
hsa-miR-93		hsa-miR-602		IVGN-novel-miR_3302
IVGN-novel-miR_3303		hsa-miR-637		IVGN-novel-miR_3379
IVGN-novel-miR_3388		hsa-miR-638		IVGN-novel-miR_3395
IVGN-novel-miR_3409		hsa-miR-663		IVGN-novel-miR_3439
IVGN-novel-miR_3440		hsa-miR-675		IVGN-novel-miR_3490
IVGN-novel-miR_3491		hsa-miR-885-3p		IVGN-novel-miR_3501
IVGN-novel-miR_3505		hsa-miR-886-5p		IVGN-novel-miR_3615
IVGN-novel-miR_3506		hsa-miR-939		IVGN-novel-miR_3616
IVGN-novel-miR_3508		IVGN-novel-miR_3476		IVGN-novel-miR_3617
IVGN-novel-miR_3528		IVGN-novel-miR_3545		IVGN-novel-miR_3618
IVGN-novel-miR_3544		IVGN-novel-miR_3546		IVGN-novel-miR_3619
IVGN-novel-miR_3561		IVGN-novel-miR_3547		IVGN-novel-miR_3627
IVGN-novel-miR_3660		IVGN-novel-miR_3603		IVGN-novel-miR_3632
IVGN-novel-miR_3662		IVGN-novel-miR_3700		IVGN-novel-miR_3639 X
IVGN-novel-miR_3663				IVGN-novel-miR_3647

X-flagged as potentially being a larger ncRNA based on profiling of distinct RNA size fractions, as described in Methods S4 in File S1.

Analysis of microarray data from fetal samples alone was performed to determine if the gestational age (in weeks) had a significant impact on expression. After adjusting for multiple testing it was found that there were no miRNAs with significant changes in expression during the fetal time period. This may be due, in part, to the small sample sizes representing each developmental time point or to the lack of sensitivity and limited dynamic range of glass-slide microarrays. Similarly, an analysis was run on the young samples alone to determine if age (in days) had a significant impact on miRNA expression. After adjusting for multiple testing, there were no miRNAs with significant changes in expression.

Samples comprising each distinct time point were mixed to form 10 sample pools (**Table 1**), and further miRNA expression analysis was performed using the TaqMan® MicroRNA Array v2.0. Many of the validated, Sanger miRNAs that were detected and quantified on TaqMan arrays fell below the threshold of detection on the NCode microarrays. Therefore the TaqMan data only partially validated the NCode array data, but also allowed for analysis of other miRNAs and for more refined analysis of expression changes within the pre- and post-natal time points. All raw real-time qPCR data is presented in **Table S1 in File S1**.

The numbers of miRNAs with particular expression fold changes between groups are presented in **Table 3**. Similar to what was observed in the microarray data set, these data demonstrate that global miRNA expression is lower in fetal than young and adult brain tissue, given the greater number of miRNAs with decreased fold change in the fetal versus young and adult samples. Although pooling of samples precludes a wide-range of formal statistical analyses, we sought to identify meaningful patterns of change within the pre- and post-natal time points. MiRNAs which show peaks of expression at a particular gestational or post-gestational time point may be viable candidate miRNAs involved in specific developmental processes. A Q–Q plot of fold changes for all miRNAs at all time points (see **Figure S2 in File S1**) revealed expected trends. After setting an arbitrary threshold to exclude miRNAs that did not show high expression in at least one time point, the standard deviations for fold changes were calculated. In analysis of pre-natal time points, miRNAs were identified which showed a >2 standard deviation fold change at any time point (relative to the 14 week time point) resulting in a list of 14 miRNAs (**Table S2 in File S1**). Particular candidate miRNAs emerge from this analysis. For example, hsa-mir-449a/449b show a dramatic change in expression during 14–20 week gestation, with a peak at week 17. Another pattern is represented by 216b, which shows highest expression at week 14, then decreasing expression until it reaches a low at week 17, followed by increasing expression through weeks 18, 19, and 20. This trend for increasing expression does not extend into post-gestational timepoints, where expression is negligible.

**Table 3:** Temporal expression analysis using real-time quantitative PCR.

Fold Increase	Fetal vs. Adult	Young vs. Adult	Fetal vs. Young	Fold Decrease	Fetal vs. Adult	Young vs. Adult	Fetal vs. Young
**>2**	145	188	90	**>2**	253	113	267
**>5**	92	85	47	**>5**	136	63	130
**>10**	68	48	27	**>10**	87	49	74
**>100**	23	23	2	**>100**	21	10	7

The number of miRNAs that exceed the indicated fold difference are tabulated for each pair-wise sample type comparison.

In the individual analysis of post-natal time points, the earliest time point (5–98 days) served as the reference against the 1.5 and 4.5 year time point. Again, miRNAs with >2 SD fold change from 5–98 day time point were identified, resulting in a list of 15 miRNAs (**Table S3 in File S1**). Particular miRNAs show clear patterns of change during early childhood. For example, hsa-mir-135b shows dramatically decreasing expression during early childhood, while at prenatal time points hsa-mir-135b showed much higher but somewhat consistent expression. Mir-542-5p on the other hand, shows consistently low expression at prenatal time points, spikes at earliest childhood time point, then declines.

Examination of the pre- and postnatal time points collectively, using 14 week as reference time point, revealed 56 miRNAs with >2 SD fold change between pre- and postnatal samples (**Table S4 in File S1**). This larger list is a reflection of the more robust collective differences in miRNA expression between fetal stages and early childhood, as compared to the more subtle changes within the fetal and early childhood periods. This analysis reveals interesting candidates, including the mir-17-92 cluster located on chromosome 13, and the paralogue clusters on chromosome 7 (mir-25/93/106b) and the X chromosome (mir-106a/18b/20b). Collectively, this miRNA family shows a trend for modestly decreasing expression at prenatal time points, and dramatically lower and decreasing expression at postnatal time points. In contrast, mir-132 displays modest and steady expression levels during prenatal time points, with a striking increase in expression during early childhood.

## Discussion

We reported 312 out of 464 detected features with temporally distinct expression patterns in the cerebrum using microarray technologies. Additionally, we presented comprehensive miRNA expression profiles of pooled samples at each time point using TaqMan technology. Utilizing the microarray data, we found that differentially expressed species formed ten unique model classes with contrasting fetal, young, and adult expression profiles. Notably, many miRNAs fit a model that did not depict a consistent trend in expression along the fetal to adult timeline, but instead sharply increased or decreased at early postnatal stages. For example, the ubiquitously expressed let-7 family members are conserved from nematodes to primates, and are well-known markers of a terminally differentiated state [22], [23], [24]. In our study, expression levels of most let-7 family members dramatically peaked at the adult time point, as expected, but we also observed a surprising decline in expression at early postnatal stages relative to fetal. We would also like to draw attention to several miRNAs with well-characterized roles in CNS development. miR-124 and miR-9 are brain-specific miRNAs [25] that are highly up-regulated at the onset of embryonic neurogenesis. Over-expression of these miRNAs in cultured cells promotes adaptation of a neuronal fate, while down-regulation has the opposite effect [26]. Developmental expression profiling of the murine CNS revealed 12 miRNAs (miR-9, miR-17-5p, miR-124a, miR-125a, miR-125b, miR-130a, miR-140, miR-181a, miR-199a, miR-205, miR-214, miR-301) with significantly higher expression at embryonic versus postnatal time points. Expression profiling was not performed in the fully mature mouse. In the present study, 4 miRNAs (miR-9, miR-124, miR-125b, miR-181a) fit the model F = A>Y. The decrease in expression at early postnatal stages is consistent with observations in the mouse; however, expression levels appear to rebound in the adult human brain. It is intriguing to note that, with regard to several neuronal miRNAs as well as the let-7 family, the early postnatal brain appears to adopt a more immature, dedifferentiated state. However, we did not observe expression of the pluripotency markers miR-290, -295, -293, -294, or the -302 family [27] at any developmental time point. With the exception of certain neurogenic brain regions, such as the dentate gyrus of the hippocampus [28], neurogenesis is mostly complete by the 18^th^ week of gestation. Our data support a role for miRNAs in later stages of neuronal development, such as the formation and pruning of synapses [29], which continues throughout infancy and early childhood. At these developmental stages, we observe transient down-regulation of several well-characterized neuronal miRNAs, with concomitant spikes in expression of 31 other Sanger miRNAs (model classes 5 and 9).

We investigated 373 novel, putative miRNAs that are exclusively present on the NCode Human miRNA microarray V3 (Invitrogen), and found that 194 of these exhibited significant temporal expression changes. Sequences were mapped to genomic hairpins and validated by qPCR with probes for the “mature” region. Subsequent bioinformatic analysis was performed using the miRDeep algorithm, which evaluates enrichment of sequence reads derived from the stem versus loop region of genomic hairpins, suggestive of miRNA processing [30]. This analysis revealed that most of the small RNA sequences were unlikely to be classic miRNAs. By comparing expression profiles of various RNA size fractions, we determined that a significant proportion of the novel sequences were likely derived from larger non-coding RNAs. Several classes of ncRNA are dynamically regulated during development, and they may serve especially prominent roles in the developing brain (reviewed in [31]). For these reasons, all microarray features were retained for significance analysis, regardless of whether they represented miRNAs or other types of RNA. Model classes 1 (F<Y = A) and 7 (F<Y<A) are comprised of 67 and 10 genes, respectively. Of these 77 genes with lowest expression during fetal stages, 74 are novel ncRNAs. From our RNA size fractionation experiments, 29 of the 312 genes with significant developmental changes in expression level were flagged as potentially being larger ncRNAs, and 26 of these flagged species were novel ncRNAs from either model class 1 (F<Y<A) or 3 (F = Y<A). Taken together, these data implicate a class of longer ncRNAs that are progressively up-regulated during development and may serve important neurobiological functions in the adult brain.

TaqMan expression profiling was more sensitive than the glass-slide microarray technology which we used, and allowed us to examine trends within the prenatal and postnatal time points. Several miRNAs were identified which have noteworthy developmental expression patterns. For example, the mir-17-92 cluster on chromosome 13 and paralogue clusters on chromosome 7 and the X chromosome show decreasing expression throughout pre- and post-natal timepoints, consistent with a report in the porcine developing brain [32]. Mir-132 shows a marked increase in post-natal compared to pre-natal time points, and appears to increase throughout early childhood. In vivo experiments in the mouse hippocampus have implicated mir-132 in the maturation of excitatory synapses formed by newborn neurons [33], and mir-132 dysregulation has recently been implicated in schizophrenia [34]. Mir-449a/b show a striking expression pattern during pre-natal development, with a peak of expression at week 17. Mir-449 has been shown to control ciliogenesis in the human airway epithelium [35], and has been proposed to regulate choroid plexus development based on expression patterns in the mouse brain [36]. The peak in mir-449 expression at week 17 may be related to ciliogenesis in the ventricular system, which if impaired, can impact ion transport and cerebrospinal fluid production and flow [37].

In summary, we have established the first miRNA and non-coding RNA expression atlas of the developing human brain. Expression signatures varied significantly between fetal, early postnatal, and adult brain tissue samples, analogous to patterns of temporal regulation previously observed in animal model systems. Despite attempts to match samples with respect to demographic variables, factors pertaining to sample selection and handling can skew the results of post-mortem expression studies. Experimental and statistical methods were adopted to optimize sensitivity and accuracy of measured miRNA expression levels, yet some caveats still remain. The coronal sections of cerebrum from which RNA was extracted are heterogeneous in terms of cell type. Expression levels were averaged across multiple samples from a given developmental age to minimize regional effects, but the relative contribution of specific cellular populations to detected expression levels could conceivably vary across time points. Also, average post-mortem interval was higher for postnatal compared to prenatal samples (**Table 1**), however our previous work demonstrates that few microRNAs (8%) have expression which is influenced by post-mortem interval [38]. Additionally, the pre- and postnatal sample set used is biased towards African-American individuals while the adult timepoints were from Caucasian individuals, which could conceivably confound results if there are large ethnic differences in microRNA expression in the brain. However, data from other tissues indicate that ethnic differences are likely to be modest [39]. Despite these caveats, the valuable expression data presented here and the proposed classification scheme should guide future studies of miRNA expression and function in both normal and pathogenic development.

## Supporting Information

File S1 (DOCX)
