# Supplementary material for: Chronological Changes in MicroRNA Expression in the Developing Human Brain
Source: PLoS One. 2013 Apr 16;8(4):e60480. doi: 10.1371/journal.pone.0060480 (PMC3628885; doi:10.1371/journal.pone.0060480)
Supplement: File S1 — (DOCX) [file pone.0060480.s001.docx]

**Supplementary Information**

**Methods S1. Merging 10% and 100% microarray scans**

There were striking differences in signal intensity between novel and validated miRNAs present on the array, and data capture was maximized by scanning all microarrays at 10% and 100% power and subsequently merging these data sets. The data was visually of high quality, and after normalization, the 100% scan values for each spot followed a linear regression model on 10% scan data in the entire range of observed log ratios. The multiple R-squared of this regression model was 93% (meaning there was a very tight correlation between the 100% and 10% scan data). Merging the datasets reduced the number of missing value spots by 50% compared with the 10% and 100% datasets analyzed separately.

Briefly, the datasets were merged as follows: The 100% scan values for each spot followed a linear regression model on 10% scan data, with the slope of the regression model found to be statistically equal to 1, and with an intercept of 0.18 (a constant shift between log ratio values in the 100% and 10% scan data). For spots where only the 10% scan data value was recorded, this value was used in the analysis. For spots where only the 100% scan data value was recorded, this value with 0.18 subtracted was used (rendering the 10% and 100% scan values directly comparable). For spots where both 10% and 100% scan values were recorded, the analysis was based on the average of the 10% and 100% scan value with 0.18 subtracted. The rationale was that by taking an average this would further suppress scanning noise in the data.

The final gene set consisted of the following groups; (1) After merging there were a total of 26 genes for which only 10% scan data was available, and among these 26 only 16 genes passed the filtering test (see Methods); (2) There were 273 genes for which only 100% scan data was available, and none of these genes passed the filtering test; (3) There were 666 genes for which both 10% and 100% data were available, and among these 448 passed the filtering test. By merging the 10% and 100% scan, the gene set that passed the filter contained 100 additional genes that would have otherwise been excluded from the analysis. Thus, merging scan data provided substantial gains in data quality (by averaging of the two scan values) and quantity.

**Methods S2. Checks of distribution assumptions**

The within-sample type variance was computed for each gene. It was found that an equal variance assumption for the different sample types was well supported by the data. Thus, ANOVA (analysis of variance, F-tests) and regression models can be used to analyze the data. A graphical examination of the error distribution using normal Quantile-Quantile plots revealed that the error distribution was long-tailed. To perform testing we thus used a re-sampling based approach to compute p-values. A model was fit to each gene where a separate mean expression level was estimated for each of the three sample types. The residuals from this fit were stored in a data matrix. We then used re-sampling techniques to generate a data set for which the null hypothesis of no sample type differences is true. This is done by randomly assigning residuals to each of the sample types, letting these re-sampled residuals take on the role of real data. Because of the random assignment the null hypothesis is true for re-sampled data. The benefit of this approach is that it is not necessary to assume a normal error distribution.

**Methods S3. Modeling expression differences between fetal, young, and adult timepoints**

Initially, all genes are modeled in a “full model”, which allows for a unique mean expression level for each of the three sample types (fetal, young, adult). The second step of the analysis is to simplify the full model. There are three models that constitute a simplification of the full model; (1) a model where the young and adult samples have the same mean expression, but the fetal samples have a different mean expression (F≠Y=A); (2) a model where the fetal and young mean expression coincides, and the adult mean expression differs (F=Y≠A); (3) a model where the fetal and adult mean expression coincides, and the young mean expression differs (F=A≠Y). These 3 models are compared in terms of fit for each gene, and the model with the smallest error sum of squares is chosen. The third step of the analysis is to compare the best model (one of models 1, 2 and 3) to the “null model” where the mean expression values for fetal, young and adult samples coincide.

In standard backward ANOVA (F-test) model selection we do the following; we fit the full model as above, and then fit models 1, 2, and 3 to the data. We perform an F-test comparing the full model to the best of models 1, 2 and 3. If this F-test leads to a rejection (the p-value is below some cutoff, e.g. 1%) we keep the full model to describe our gene. If, on the other hand, the F-test leads to a non-rejection, we select the best of models 1, 2 and 3 to describe the gene. We then perform an F-test comparing the best of models 1, 2 and 3 to the null model. If the p-value of this test is below the cutoff 1%, we keep the best of models 1, 2 and 3. If the p-value of this test is above the cutoff 1%, we keep the null model to describe our gene.

**Methods S4. Microarray analysis of distinct RNA fractions**

Traditionally, miRNA microarrays have required the use of total RNA fractions enriched for small RNA in order to ensure robust and specific detection of miRNAs. However, the NCode V3 array is compatible with the use of total RNA (as recommended by the manufacturer). To avoid potentially biasing expression profiles due to size fractionation, total RNA was used in all final microarray experiments. However, due to the striking signal intensity observed for many of the novel microRNAs present on the NCode V3 array, we questioned whether these signals indicated hybridization of miRNA precursors or other large non-coding RNA species, rather than mature miRNAs. oHTo test this possibility, miRNA expression arrays were performed using small and large RNA size fractions. Distinct RNA fractions were prepared from total RNA (First Choice Human Brain, Ambion), which were either depleted of or enriched for small RNAs using *mir*Vana (Ambion) glass-fiber filter based protocols. This process should leave a small RNA fraction containing mostly RNA species less than 200bp and a large RNA fraction containing mostly RNA species of greater than 200bp.Small and large RNA fractions were independently labeled and hybridized to NCode V3 arrays as previously described. For all spotted features, we calculated the difference in background subtracted median fluorescence between the large and small RNA fractions. The expectation is that little specific signal (derived from mature miRNAs) should be seen in the large RNA fraction. We compared background subtracted median fluorescence values from small and large RNA fractions (**Figure SF1**). The expectation is that for probes specific to mature microRNAshigher signal will be observed when hybridizing the small RNA fraction. Indeed, only a handful of traditional (Sanger) miRNAs violated this expectation. However, a much greater proportion of the novel, putative miRNAs showed higher signal in the large RNA fraction, implying that these constitute RNAs of greater than 200bp. These species were not excluded from the statistical analysis, but they were flagged as potentially belonging to other classes of non-coding RNA (**Table 2**).

Additionally, we compared alternate labeling methods (enzymatic and chemical) as well as alternate hybridization temperatures (data not shown). While the labeling method did not significantly affect the sensitivity and specificity of the assay, hybridization temperature clearly did. We chose to pursue a lower hybridization temperature than that recommended by the manufacturer, which resulted in more false positives but far fewer false negatives as determined by experiments using synthetic pools of miRNAs (data not shown).


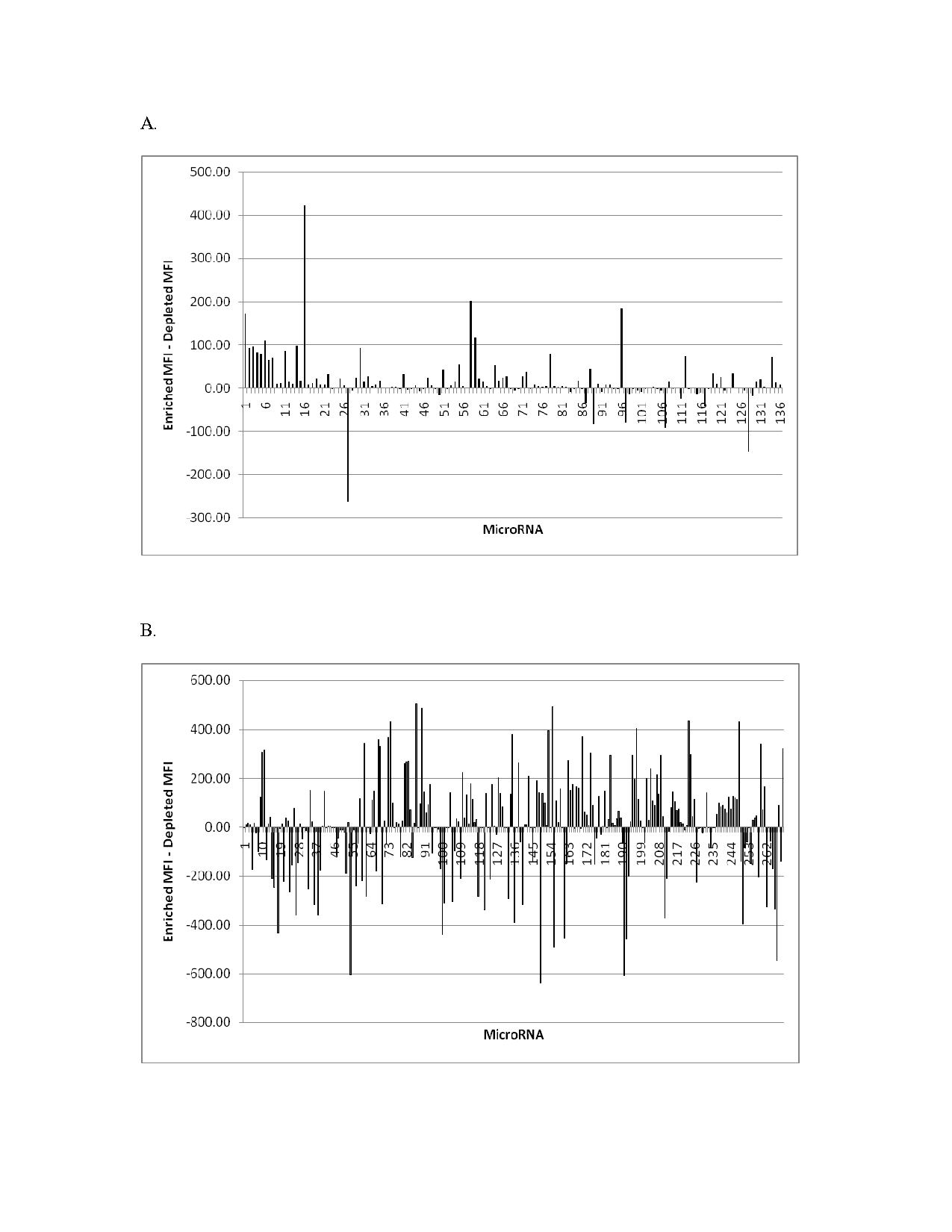
**Figure S1: Expression analysis of size fractionated RNA samples.** Total RNA samples were either enriched for or depleted of small RNAs. 1 μg each of enriched and depleted samples were labeled and hybridized to glass slide arrays. The background subtracted median fluorescence (MFI) was calculated, and the MFI of depleted samples was subtracted from the MFI of enriched samples. A) represents detectable Sanger miRNAs, B) represents detectable novel miRNAs.

**Table S1. Raw TaqMan expression data from pooled timepoints**

| detector | 14 week | 16 week | 17 week | 18 week | 19 week | 20 week | 5-98 days | 443-502 days | 1630-1733 days |
| --- | --- | --- | --- | --- | --- | --- | --- | --- | --- |
| ath-miR159a-4373390 | 40 | 40 | 40 | 40 | 40 | 40 | 40 | 40 | 40 |
| has-miR-155-4395459 | 36.99 | 36.19 | 40 | 35.45 | 35.35 | 40 | 40 | 34.51 | 34.25 |
| hsa-let-7a*-4395418 | 33.13 | 31.98 | 32.06 | 33.04 | 32.25 | 33.02 | 32.89 | 40 | 36 |
| hsa-let-7a-4373169 | 27.93 | 27.99 | 27.86 | 27.59 | 26.96 | 27.34 | 27.98 | 28.51 | 28.33 |
| hsa-let-7b*-4395515 | 33.12 | 32.93 | 36.04 | 32.29 | 40 | 40 | 40 | 36.83 | 36.68 |
| hsa-let-7b-4395446 | 24.8 | 24.4 | 24.45 | 24.58 | 23.93 | 24.01 | 25.12 | 25.97 | 25.8 |
| hsa-let-7c-4373167 | 27.74 | 26.97 | 27.24 | 26.82 | 26.38 | 26.67 | 28.06 | 28.74 | 28.65 |
| hsa-let-7d*-4378108 | 40 | 32.63 | 40 | 40 | 40 | 40 | 40 | 33.24 | 40 |
| hsa-let-7d-4395394 | 27.95 | 28.22 | 28.42 | 28.32 | 28.19 | 28.54 | 28.99 | 28.8 | 28.25 |
| hsa-let-7e*-4395518 | 30.87 | 32.63 | 31.09 | 30.91 | 36.03 | 34.99 | 40 | 31.49 | 34.48 |
| hsa-let-7e-4395517 | 24.96 | 24.96 | 24.98 | 24.88 | 24.12 | 24.63 | 25.2 | 25.83 | 25.68 |
| hsa-let-7f-1*-4395528 | 33.47 | 33.8 | 35.98 | 40 | 40 | 40 | 40 | 40 | 40 |
| hsa-let-7f-2*-4395529 | 33.67 | 34.83 | 34.14 | 32.64 | 30.32 | 32.69 | 35.97 | 35.19 | 31.31 |
| hsa-let-7f-4373164 | 28.04 | 28.05 | 27.87 | 27.67 | 26.77 | 27.93 | 28.3 | 28.46 | 27.76 |
| hsa-let-7g*-4395229 | 31.02 | 31.13 | 31.68 | 30.84 | 30.5 | 31.92 | 32.96 | 31.6 | 31.48 |
| hsa-let-7g-4395393 | 26.91 | 26.78 | 26.65 | 26.44 | 26.02 | 26.47 | 27.45 | 27.56 | 26.95 |
| hsa-let-7i*-4395283 | 33.37 | 32.48 | 32.73 | 32.88 | 32.99 | 32.46 | 34.83 | 34.98 | 33.47 |
| hsa-miR-100*-4395253 | 31.24 | 30.77 | 30.79 | 30.73 | 30.64 | 30.48 | 35.01 | 34.08 | 35.64 |
| hsa-miR-100-4373160 | 25.84 | 25.14 | 24.95 | 24.96 | 24.49 | 24.55 | 26.46 | 26.54 | 26.06 |
| hsa-miR-101*-4395254 | 31.7 | 30.37 | 31.11 | 30.49 | 31.16 | 30.98 | 33.14 | 34.71 | 31.66 |
| hsa-miR-101-4395364 | 30.37 | 30.38 | 30.12 | 29.94 | 29.64 | 30.3 | 31.4 | 31.02 | 30.87 |
| hsa-miR-103-4373158 | 26.68 | 27.62 | 27.49 | 27.25 | 27.02 | 27.04 | 27.66 | 27.7 | 27 |
| hsa-miR-105*-4395279 | 40 | 40 | 40 | 40 | 35.05 | 40 | 40 | 40 | 40 |
| hsa-miR-105-4395278 | 31 | 32.27 | 31.23 | 30.19 | 31.24 | 30.29 | 31.66 | 32.35 | 33.93 |
| hsa-miR-106a*-4395281 | 34.01 | 40 | 40 | 40 | 40 | 40 | 40 | 40 | 35.12 |
| hsa-miR-106a-4395280 | 21.51 | 22 | 21.8 | 21.85 | 21.61 | 21.96 | 25.31 | 25.92 | 26.14 |
| hsa-miR-106b*-4395491 | 26.42 | 27.81 | 27.38 | 27.81 | 27.25 | 28.3 | 35.35 | 33.67 | 35.92 |
| hsa-miR-106b-4373155 | 25.36 | 25.88 | 25.8 | 25.9 | 25.44 | 25.82 | 28.62 | 28.96 | 28.85 |
| hsa-miR-107-4373154 | 29.64 | 30.35 | 30.19 | 29.59 | 31.01 | 30.3 | 30.82 | 31.3 | 30.23 |
| hsa-miR-10a*-4395399 | 32.76 | 33.11 | 31.83 | 35.86 | 40 | 34.9 | 35.15 | 32.56 | 40 |
| hsa-miR-10a-4373153 | 31.83 | 31.15 | 31.47 | 30.51 | 30.94 | 30.13 | 30.99 | 32.66 | 31.8 |
| hsa-miR-10b*-4395426 | 31.19 | 34.3 | 33.13 | 36.01 | 33.14 | 31.62 | 34.96 | 35.16 | 33.84 |
| hsa-miR-10b-4395329 | 40 | 40 | 40 | 40 | 40 | 40 | 40 | 40 | 35.92 |
| hsa-miR-122-4395356 | 31.91 | 28.62 | 40 | 32.6 | 30.74 | 36.39 | 31.97 | 40 | 40 |
| hsa-miR-124*-4395308 | 26.91 | 26.99 | 26.96 | 26.91 | 26.73 | 26.66 | 29.11 | 29.44 | 28.45 |
| hsa-miR-124-4373295 | 24.96 | 24.24 | 24.31 | 24.13 | 23.8 | 23.54 | 24.73 | 24.85 | 24.69 |
| hsa-miR-125a-3p-4395310 | 32.77 | 33.99 | 33.4 | 33.71 | 33.32 | 33.03 | 33.92 | 34.34 | 35.67 |
| hsa-miR-125a-5p-4395309 | 26.95 | 27 | 26.64 | 26.88 | 26.47 | 26.53 | 27.66 | 27.83 | 27.58 |
| hsa-miR-125b-1*-4395489 | 27.68 | 27.69 | 27.86 | 27.75 | 27.52 | 27.92 | 30.76 | 31.37 | 31.47 |
| hsa-miR-125b-2*-4395269 | 30.09 | 27.77 | 28.08 | 27.67 | 26.95 | 28.12 | 31.54 | 31.66 | 29.86 |
| hsa-miR-125b-4373148 | 23.21 | 22.58 | 22.57 | 22.53 | 21.98 | 22.11 | 24.24 | 24.6 | 24.21 |
| hsa-miR-126*-4373269 | 29.35 | 29.26 | 28.97 | 28.48 | 28.45 | 28.7 | 28.38 | 27.58 | 27.52 |
| hsa-miR-126-4395339 | 24.87 | 24.96 | 24.86 | 24.28 | 24.29 | 24.4 | 23.45 | 23.46 | 22.95 |
| hsa-miR-127-3p-4373147 | 28.88 | 29.1 | 29.25 | 28.29 | 28.87 | 28.94 | 28.62 | 27.99 | 27.75 |
| hsa-miR-127-5p-4395340 | 34.04 | 31.49 | 31.53 | 34.2 | 31.4 | 31.34 | 30.81 | 32.04 | 29.67 |
| hsa-miR-128-4395327 | 28.36 | 26.91 | 26.95 | 26.62 | 26.23 | 26.14 | 26.38 | 26.72 | 25.95 |
| hsa-miR-129-3p-4373297 | 29.22 | 29.98 | 29.75 | 28.94 | 29.58 | 29.65 | 28.97 | 28.19 | 27.81 |
| hsa-miR-129-5p-4373171 | 31.89 | 32.53 | 31.56 | 32.97 | 31.23 | 33.29 | 29.77 | 30.11 | 30.26 |
| hsa-miR-130a*-4395242 | 35.05 | 40 | 40 | 32.61 | 30.54 | 32.79 | 40 | 34.83 | 35.71 |
| hsa-miR-130a-4373145 | 27.44 | 28.49 | 27.97 | 27.97 | 27.67 | 27.83 | 30.77 | 31.85 | 31.37 |
| hsa-miR-130b*-4395225 | 26.45 | 27.3 | 27.26 | 27.37 | 26.73 | 27.26 | 31.42 | 32.67 | 31.81 |
| hsa-miR-130b-4373144 | 25.68 | 26.91 | 26.93 | 26.7 | 26.66 | 26.99 | 30.92 | 30.95 | 30.44 |
| hsa-miR-132*-4395243 | 40 | 34.03 | 40 | 40 | 40 | 40 | 31.06 | 30.91 | 30 |
| hsa-miR-132-4373143 | 26.81 | 26.45 | 26.22 | 25.94 | 25.97 | 26.11 | 23.35 | 23.25 | 22.69 |
| hsa-miR-133a-4395357 | 35.86 | 32.96 | 33.05 | 31.87 | 32.81 | 33.91 | 30.95 | 29.56 | 28.78 |
| hsa-miR-133b-4395358 | 40 | 33.78 | 40 | 32.95 | 32.15 | 32.76 | 31.77 | 31.01 | 30.4 |
| hsa-miR-134-4373299 | 29.95 | 29.44 | 29.43 | 29.14 | 29.66 | 30.03 | 29.01 | 29.24 | 29.13 |
| hsa-miR-135a*-4395343 | 28.58 | 27.96 | 28.09 | 28.38 | 28.23 | 28.16 | 28.51 | 27.95 | 28.28 |
| hsa-miR-135a-4373140 | 27.35 | 26.98 | 27.01 | 26.42 | 26.89 | 27.96 | 28.3 | 29.96 | 29.69 |
| hsa-miR-135b*-4395270 | 26.41 | 27.77 | 27.3 | 27.29 | 27.59 | 27.25 | 32.81 | 36.43 | 32.45 |
| hsa-miR-135b-4395372 | 23.96 | 24.24 | 24.24 | 23.99 | 23.82 | 24.13 | 27.74 | 30.46 | 31.8 |
| hsa-miR-136*-4395211 | 31.43 | 31.44 | 31.26 | 30.46 | 30.97 | 31.28 | 30.31 | 29.77 | 29.29 |
| hsa-miR-136-4373173 | 32.41 | 33.74 | 33.05 | 33.33 | 33.16 | 35.11 | 33.85 | 37.42 | 34.27 |
| hsa-miR-137-4373301 | 27.7 | 26.4 | 25.96 | 25.76 | 25.32 | 25.25 | 26.25 | 26.62 | 27.17 |
| hsa-miR-138-1*-4395273 | 32.41 | 32 | 31.7 | 32.1 | 32.7 | 32.1 | 32.61 | 31.76 | 31.79 |
| hsa-miR-138-2*-4395255 | 30.46 | 31.49 | 31.39 | 30.22 | 30.06 | 31.5 | 32.05 | 31.15 | 30.18 |
| hsa-miR-138-4395395 | 29.26 | 27.7 | 27.43 | 26.55 | 26.6 | 26.66 | 26.17 | 25.96 | 25.96 |
| hsa-miR-139-3p-4395424 | 28.73 | 29.02 | 28.61 | 27.96 | 28.13 | 28.01 | 26.91 | 26.49 | 25.75 |
| hsa-miR-139-5p-4395400 | 25.87 | 26.26 | 25.97 | 25.48 | 25.59 | 25.7 | 24.57 | 24.2 | 23.95 |
| hsa-miR-140-3p-4395345 | 29.87 | 30.32 | 29.69 | 29.99 | 29.12 | 29.83 | 30.63 | 29.97 | 30.35 |
| hsa-miR-140-5p-4373374 | 26.7 | 26.91 | 26.55 | 26.49 | 25.85 | 26.15 | 27.37 | 26.95 | 26.6 |
| hsa-miR-141*-4395256 | 40 | 40 | 40 | 40 | 40 | 37.62 | 40 | 40 | 40 |
| hsa-miR-141-4373137 | 33.88 | 32.46 | 34.03 | 35.53 | 33.95 | 36.94 | 40 | 34.01 | 35.12 |
| hsa-miR-142-3p-4373136 | 31.97 | 32.27 | 31.83 | 31.69 | 32.36 | 32.15 | 30.2 | 29.53 | 30.58 |
| hsa-miR-142-5p-4395359 | 33.68 | 40 | 40 | 33.93 | 40 | 35.6 | 32.84 | 33.12 | 32.96 |
| hsa-miR-143*-4395257 | 40 | 40 | 40 | 40 | 40 | 40 | 40 | 40 | 40 |
| hsa-miR-143-4395360 | 29.94 | 30.53 | 30.98 | 29.18 | 29.46 | 29.09 | 28.77 | 28.64 | 28.26 |
| hsa-miR-1-4395333 | 30.95 | 30.47 | 32.08 | 31.31 | 31.29 | 31.97 | 29.12 | 28.95 | 29.08 |
| hsa-miR-144*-4395259 | 33.8 | 32.82 | 32.29 | 33.96 | 33.97 | 33.67 | 31.93 | 32.55 | 33.21 |
| hsa-miR-145*-4395260 | 37 | 31.84 | 31.57 | 33.53 | 31.84 | 32.5 | 32.62 | 32.44 | 31.09 |
| hsa-miR-145-4395389 | 28.97 | 28.92 | 28.89 | 28.17 | 28.59 | 28.3 | 27.83 | 27.5 | 27.01 |
| hsa-miR-146a*-4395274 | 40 | 40 | 36 | 40 | 40 | 40 | 40 | 40 | 40 |
| hsa-miR-146a-4373132 | 30.42 | 29.52 | 29.18 | 28.71 | 28.97 | 29.32 | 27.39 | 27.54 | 27.49 |
| hsa-miR-146b-3p-4395472 | 40 | 40 | 40 | 40 | 34.02 | 40 | 40 | 40 | 32.63 |
| hsa-miR-146b-5p-4373178 | 30.4 | 29.95 | 30.03 | 29.72 | 29.26 | 29.17 | 27.96 | 26.52 | 26.12 |
| hsa-miR-147-4373131 | 36.01 | 40 | 40 | 40 | 40 | 40 | 40 | 40 | 40 |
| hsa-miR-147b-4395373 | 40 | 40 | 40 | 40 | 40 | 40 | 40 | 40 | 40 |
| hsa-miR-148a*-4395245 | 28.97 | 40 | 40 | 31.14 | 40 | 30.87 | 40 | 40 | 29.65 |
| hsa-miR-148a-4373130 | 31.64 | 32.41 | 32.08 | 31.97 | 30.95 | 30.48 | 30.59 | 31.72 | 32.83 |
| hsa-miR-148b*-4395271 | 32.72 | 34.86 | 33.02 | 35.01 | 33.98 | 33.46 | 36.63 | 33.6 | 33.74 |
| hsa-miR-148b-4373129 | 30.31 | 30.95 | 31.23 | 31.11 | 30.92 | 31.53 | 32.46 | 32.29 | 31.76 |
| hsa-miR-149*-4395275 | 26.77 | 26.19 | 26.24 | 26.49 | 26.74 | 26.45 | 26.63 | 26.87 | 26.96 |
| hsa-miR-149-4395366 | 23.73 | 23.95 | 23.97 | 23.87 | 23.38 | 23.54 | 25.27 | 24.95 | 24.82 |
| hsa-miR-150-4373127 | 30.81 | 30.95 | 30.32 | 29.53 | 29.77 | 30.14 | 28.53 | 28.39 | 28.07 |
| hsa-miR-151-3p-4395365 | 25.72 | 26.91 | 26.7 | 26.74 | 26.67 | 26.92 | 27.92 | 27.1 | 27.14 |
| hsa-miR-152-4395170 | 30.9 | 31.98 | 31.95 | 30.43 | 30.84 | 31 | 30.24 | 29.81 | 29.7 |
| hsa-miR-153-4373305 | 33.8 | 31.76 | 31.45 | 30.91 | 30.75 | 31.42 | 31.02 | 31.96 | 31.61 |
| hsa-miR-154*-4378065 | 40 | 33.45 | 35.99 | 40 | 32.28 | 34.21 | 40 | 33.83 | 30.91 |
| hsa-miR-154-4373270 | 33.75 | 33.72 | 33.49 | 31.64 | 40 | 40 | 36.02 | 33.61 | 33.26 |
| hsa-miR-155*-4395398 | 40 | 40 | 40 | 40 | 40 | 40 | 36.97 | 40 | 40 |
| hsa-miR-15a*-4395530 | 30.74 | 31.27 | 30.54 | 30.62 | 30.78 | 31.71 | 32.97 | 33.1 | 34.96 |
| hsa-miR-15a-4373123 | 26.99 | 26.93 | 27.16 | 26.81 | 26.47 | 27.5 | 29.42 | 29.76 | 29 |
| hsa-miR-15b*-4395284 | 24.73 | 25.89 | 26.18 | 26.04 | 25.58 | 26.84 | 31.26 | 32.1 | 30.65 |
| hsa-miR-15b-4373122 | 25.98 | 26.91 | 26.73 | 26.96 | 26.4 | 27.46 | 30.06 | 30.64 | 30.54 |
| hsa-miR-16-1*-4395531 | 29.93 | 30.03 | 30.33 | 31.55 | 30.27 | 30.8 | 40 | 40 | 31.7 |
| hsa-miR-16-2*-4395282 | 40 | 40 | 40 | 40 | 40 | 33.24 | 40 | 40 | 40 |
| hsa-miR-16-4373121 | 22.7 | 22.78 | 22.5 | 22.3 | 22.13 | 22.96 | 25.26 | 25.35 | 25.27 |
| hsa-miR-17*-4395532 | 27.65 | 27.97 | 27.68 | 27.5 | 27.78 | 28.55 | 36.16 | 33.24 | 31.7 |
| hsa-miR-17-4395419 | 21.15 | 21.62 | 21.33 | 21.43 | 21.14 | 21.64 | 24.93 | 25.66 | 25.95 |
| hsa-miR-181a*-4373086 | 28.1 | 27.98 | 27.86 | 27.51 | 27.45 | 27.55 | 29.76 | 29.97 | 29.35 |
| hsa-miR-181a-2*-4395428 | 28.05 | 28.52 | 28.64 | 28.84 | 28.31 | 29.42 | 31.25 | 31.98 | 31.69 |
| hsa-miR-181a-4373117 | 25.66 | 24.96 | 24.62 | 24.58 | 23.69 | 23.95 | 25.64 | 25.96 | 25.96 |
| hsa-miR-181c*-4395444 | 28.7 | 27.96 | 27.77 | 27.91 | 27.41 | 27.91 | 29.56 | 30.32 | 29.38 |
| hsa-miR-181c-4373115 | 28.56 | 27.8 | 27.71 | 28 | 27.53 | 28.29 | 29.78 | 30.4 | 30.96 |
| hsa-miR-182-4395445 | 38.27 | 30.78 | 32.6 | 32.13 | 29.63 | 35.16 | 33.47 | 37.16 | 40 |
| hsa-miR-183*-4395381 | 32.76 | 32.88 | 34.97 | 31.68 | 40 | 36.36 | 35.3 | 40 | 34.72 |
| hsa-miR-183-4395380 | 34.65 | 40 | 32.32 | 35.02 | 40 | 40 | 40 | 40 | 40 |
| hsa-miR-184-4373113 | 37.08 | 39.42 | 33.48 | 32.34 | 36.74 | 32.69 | 30.56 | 30.73 | 30.58 |
| hsa-miR-185-4395382 | 29.98 | 30.53 | 30.7 | 30.55 | 29.96 | 30.24 | 30.29 | 28.98 | 29.44 |
| hsa-miR-186*-4395216 | 29.87 | 31.32 | 32.04 | 30.89 | 30.43 | 32.05 | 40 | 40 | 40 |
| hsa-miR-186-4395396 | 27.84 | 28.58 | 27.97 | 27.96 | 27.24 | 27.89 | 29.63 | 29.24 | 29.26 |
| hsa-miR-187-4373307 | 32.26 | 31 | 32 | 30.18 | 29.91 | 33.19 | 28.33 | 27.95 | 28.74 |
| hsa-miR-188-3p-4395217 | 40 | 40 | 40 | 40 | 40 | 40 | 40 | 40 | 31.05 |
| hsa-miR-188-5p-4395431 | 30.39 | 29.62 | 30.13 | 30.58 | 30.34 | 30.52 | 30.48 | 31.01 | 31.43 |
| hsa-miR-18a*-4395534 | 29.81 | 31.7 | 31.35 | 31.94 | 32.16 | 33.97 | 40 | 35.08 | 40 |
| hsa-miR-18a-4395533 | 27.64 | 28.58 | 28.62 | 28.91 | 28.53 | 28.89 | 31.95 | 32.35 | 32.42 |
| hsa-miR-18b*-4395421 | 40 | 40 | 33.19 | 40 | 40 | 40 | 40 | 40 | 40 |
| hsa-miR-18b-4395328 | 28.27 | 29.06 | 29.62 | 29.98 | 28.68 | 28.86 | 31.85 | 31.96 | 32.93 |
| hsa-miR-190-4373110 | 30.82 | 31.96 | 31.79 | 31.73 | 31.83 | 32.07 | 32.05 | 32.18 | 32.09 |
| hsa-miR-190b-4395374 | 32.12 | 33.99 | 31.3 | 32.5 | 32.09 | 32.3 | 33.39 | 32.07 | 32.71 |
| hsa-miR-191-4395410 | 24.97 | 25.4 | 24.96 | 24.95 | 24.62 | 24.82 | 25.6 | 25.1 | 24.68 |
| hsa-miR-192*-4395383 | 37 | 40 | 32.82 | 40 | 40 | 31.69 | 40 | 35.19 | 40 |
| hsa-miR-192-4373108 | 31.44 | 31.62 | 30.97 | 30.62 | 30.96 | 31.12 | 31.22 | 31.56 | 31.41 |
| hsa-miR-193a-3p-4395361 | 33.45 | 33.47 | 40 | 32.85 | 36.09 | 33.82 | 32.07 | 31.36 | 31.98 |
| hsa-miR-193a-5p-4395392 | 35.03 | 33.83 | 35.95 | 32.62 | 33.16 | 32.73 | 32.54 | 31.89 | 31.78 |
| hsa-miR-193b*-4395477 | 29.37 | 30.17 | 30.97 | 29.53 | 40 | 30.02 | 30.98 | 29.78 | 29.62 |
| hsa-miR-193b-4395478 | 29.22 | 29.9 | 29.96 | 29.1 | 29.62 | 29.21 | 29.8 | 29.27 | 29.47 |
| hsa-miR-194-4373106 | 31.57 | 31.3 | 31.8 | 31.27 | 31.34 | 31.42 | 32.2 | 31.97 | 31.93 |
| hsa-miR-195-4373105 | 26.99 | 27.02 | 26.99 | 26.66 | 26.79 | 27.21 | 26.38 | 27.04 | 26.22 |
| hsa-miR-196b-4395326 | 32.97 | 35.79 | 36.69 | 32.03 | 40 | 40 | 31.97 | 36.99 | 40 |
| hsa-miR-197-4373102 | 26.96 | 27.61 | 27.38 | 27.66 | 27.07 | 27.82 | 28.98 | 28.99 | 28.19 |
| hsa-miR-198-4395384 | 31.01 | 31.8 | 31.56 | 32.01 | 31.9 | 35.77 | 30.7 | 33.04 | 32.73 |
| hsa-miR-199a-3p-4395415 | 29.58 | 31.36 | 31.36 | 29.98 | 30 | 29.32 | 30.28 | 30.68 | 30.21 |
| hsa-miR-199a-5p-4373272 | 31.94 | 40 | 40 | 32.34 | 40 | 37.06 | 40 | 40 | 40 |
| hsa-miR-199b-5p-4373100 | 40 | 40 | 36.92 | 33.22 | 40 | 40 | 32.09 | 40 | 35.67 |
| hsa-miR-19a*-4395535 | 32.12 | 35.07 | 31.65 | 33.02 | 33.74 | 33.98 | 40 | 40 | 40 |
| hsa-miR-19a-4373099 | 21.73 | 21.96 | 21.9 | 22.25 | 22.12 | 22.74 | 25.7 | 27.27 | 27.52 |
| hsa-miR-19b-1*-4395536 | 29.3 | 29.42 | 29.46 | 29.5 | 29.04 | 29.63 | 33.99 | 33 | 34.58 |
| hsa-miR-19b-4373098 | 20.34 | 20.38 | 20.18 | 20.26 | 19.97 | 20.39 | 22.78 | 23.91 | 23.77 |
| hsa-miR-200a*-4373273 | 36 | 33.95 | 40 | 40 | 40 | 40 | 40 | 34.66 | 40 |
| hsa-miR-200a-4378069 | 31.86 | 31.59 | 31.03 | 31.44 | 31.1 | 30.72 | 31.9 | 31.64 | 31.63 |
| hsa-miR-200b-4395362 | 31.26 | 31.71 | 32.48 | 32 | 31.02 | 32.21 | 33.74 | 33.72 | 31.55 |
| hsa-miR-200c-4395411 | 31.68 | 30.98 | 31.13 | 30.99 | 30.53 | 30.84 | 35.02 | 30.69 | 31.4 |
| hsa-miR-202*-4395473 | 40 | 28.14 | 28.43 | 40 | 40 | 32.12 | 40 | 40 | 40 |
| hsa-miR-202-4395474 | 32.09 | 31.71 | 31.59 | 32.42 | 31.59 | 30.91 | 31.59 | 31.71 | 33.93 |
| hsa-miR-203-4373095 | 32.23 | 33.39 | 31.66 | 32.96 | 31.91 | 32.48 | 33.08 | 30.88 | 30.99 |
| hsa-miR-204-4373094 | 28.3 | 26.98 | 26.46 | 26.64 | 25.61 | 26.02 | 27.16 | 26.83 | 26.46 |
| hsa-miR-205-4373093 | 40 | 40 | 40 | 40 | 40 | 40 | 40 | 29.11 | 40 |
| hsa-miR-206-4373092 | 35.13 | 31.92 | 34.93 | 32.01 | 31.13 | 30.73 | 36.54 | 35.88 | 33.9 |
| hsa-miR-208-4373091 | 30.69 | 33.64 | 32.34 | 34.77 | 33.93 | 33.85 | 33.92 | 34.88 | 33.24 |
| hsa-miR-208b-4395401 | 30.63 | 30.95 | 29.56 | 29.02 | 29.97 | 28.95 | 30.55 | 29.9 | 28.96 |
| hsa-miR-20a*-4395548 | 29.34 | 29.55 | 29.36 | 29.24 | 29.14 | 29.65 | 32.63 | 33.64 | 33.7 |
| hsa-miR-20a-4373286 | 22.06 | 22.42 | 22.33 | 22.66 | 22.05 | 22.65 | 25.99 | 26.96 | 27.19 |
| hsa-miR-20b*-4395422 | 30.9 | 40 | 32.62 | 34.74 | 34.11 | 35.84 | 33.03 | 40 | 40 |
| hsa-miR-20b-4373263 | 26.73 | 27.95 | 27.87 | 27.9 | 27.91 | 28.38 | 29.9 | 29.6 | 29.44 |
| hsa-miR-21*-4395549 | 30.45 | 32.14 | 31 | 32.05 | 31.75 | 32.52 | 36 | 31.19 | 32.58 |
| hsa-miR-210-4373089 | 26.56 | 26.85 | 26.7 | 26.76 | 26.5 | 26.69 | 28.36 | 29.19 | 28.93 |
| hsa-miR-211-4373088 | 40 | 40 | 40 | 40 | 40 | 40 | 40 | 40 | 40 |
| hsa-miR-212-4373087 | 32.58 | 31.96 | 31.93 | 31.42 | 31.98 | 32.64 | 29.31 | 29.32 | 29.02 |
| hsa-miR-214*-4395404 | 34.01 | 40 | 35.97 | 32.31 | 31.91 | 30.67 | 32.04 | 31.71 | 33.16 |
| hsa-miR-21-4373090 | 27.96 | 27.94 | 27.84 | 27.56 | 27.4 | 27.85 | 28.34 | 26.6 | 28.13 |
| hsa-miR-214-4395417 | 28.82 | 29.82 | 29.99 | 29.45 | 28.37 | 28.42 | 28.89 | 29.06 | 29.49 |
| hsa-miR-215-4373084 | 31.61 | 31.78 | 30.79 | 31.66 | 30.04 | 31.53 | 30.99 | 31.01 | 32.46 |
| hsa-miR-216a-4395331 | 30.45 | 34.33 | 34.25 | 32.69 | 34 | 32.23 | 40 | 40 | 34.51 |
| hsa-miR-216b-4395437 | 27.13 | 30.82 | 31.42 | 29.66 | 29.31 | 28.98 | 40 | 32.44 | 35.88 |
| hsa-miR-217-4395448 | 31.68 | 33.75 | 35.66 | 33.97 | 33.7 | 33.77 | 40 | 40 | 40 |
| hsa-miR-218-2*-4395405 | 33.74 | 34.96 | 34.54 | 33.98 | 34.34 | 34.9 | 40 | 40 | 34.81 |
| hsa-miR-218-4373081 | 26.95 | 27.03 | 27.08 | 26.53 | 26.41 | 26.32 | 25.36 | 24.96 | 24.41 |
| hsa-miR-219-1-3p-4395206 | 28.85 | 33.72 | 32.71 | 33.12 | 31.39 | 35.93 | 40 | 40 | 40 |
| hsa-miR-219-2-3p-4395501 | 40 | 33.11 | 34.9 | 30.38 | 40 | 32.33 | 27.25 | 26.49 | 26.57 |
| hsa-miR-219-5p-4373080 | 31.53 | 31.87 | 34 | 33.88 | 33.99 | 33.04 | 29.83 | 28.37 | 27.76 |
| hsa-miR-22*-4395412 | 32.46 | 31.6 | 31.51 | 31.8 | 30.97 | 32.01 | 30.63 | 29.44 | 29.24 |
| hsa-miR-220-4373078 | 40 | 40 | 36.69 | 40 | 40 | 40 | 40 | 40 | 40 |
| hsa-miR-220b-4395317 | 33.04 | 35.33 | 33.12 | 40 | 40 | 40 | 40 | 40 | 40 |
| hsa-miR-220c-4395322 | 29.99 | 40 | 40 | 40 | 31.24 | 40 | 40 | 40 | 30.82 |
| hsa-miR-221*-4395207 | 30.43 | 36.34 | 32.34 | 30.94 | 31.85 | 30.35 | 36.72 | 30.02 | 30.46 |
| hsa-miR-221-4373077 | 25.95 | 27.4 | 27.4 | 27.37 | 27.18 | 27.48 | 28.34 | 27.79 | 27.34 |
| hsa-miR-222*-4395208 | 26.14 | 28.29 | 28.57 | 27.68 | 27.81 | 28.17 | 31.55 | 29.95 | 29.5 |
| hsa-miR-222-4395387 | 24.23 | 25.27 | 25.19 | 25.34 | 25.06 | 25.69 | 25.64 | 24.94 | 24.64 |
| hsa-miR-223*-4395209 | 30.91 | 33.22 | 31.15 | 32.35 | 31.93 | 30.76 | 31.43 | 28.93 | 30.54 |
| hsa-miR-223-4395406 | 29.95 | 29.52 | 29.67 | 28.67 | 28.6 | 28.75 | 27.24 | 25.95 | 26.65 |
| hsa-miR-22-4373079 | 27.67 | 27.75 | 27.58 | 27.07 | 27.64 | 27.94 | 25.28 | 25.26 | 24.74 |
| hsa-miR-224-4395210 | 29.52 | 31.12 | 31.76 | 30.83 | 31.37 | 31.46 | 35.97 | 33.25 | 40 |
| hsa-miR-23a*-4395550 | 31.54 | 32.25 | 36.32 | 32.28 | 36.24 | 36.3 | 34.5 | 40 | 40 |
| hsa-miR-23a-4373074 | 28.39 | 30.06 | 29.91 | 29.51 | 29.02 | 29.65 | 27.9 | 29.31 | 29.51 |
| hsa-miR-23b*-4395237 | 40 | 40 | 40 | 40 | 31.17 | 40 | 40 | 40 | 40 |
| hsa-miR-23b-4373073 | 30.56 | 30.48 | 30.06 | 30.38 | 29.94 | 32.06 | 29.57 | 29.02 | 28.46 |
| hsa-miR-24-1*-4395551 | 40 | 40 | 40 | 40 | 40 | 40 | 40 | 40 | 40 |
| hsa-miR-24-4373072 | 24.45 | 24.97 | 24.92 | 24.83 | 24.53 | 24.94 | 23.98 | 23.78 | 23.12 |
| hsa-miR-25*-4395553 | 30.92 | 29.84 | 34.74 | 32.1 | 34.51 | 32.17 | 40 | 40 | 40 |
| hsa-miR-25-4373071 | 27.62 | 28.96 | 28.94 | 29.02 | 28.68 | 29.66 | 32.74 | 32.45 | 32.77 |
| hsa-miR-26a-1*-4395554 | 34.02 | 33.86 | 33.23 | 33.3 | 32.62 | 33.6 | 33.59 | 33.47 | 33.97 |
| hsa-miR-26a-2*-4395226 | 31.65 | 32.91 | 32.29 | 32.16 | 31.6 | 33.54 | 36.96 | 40 | 40 |
| hsa-miR-26a-4395166 | 23.76 | 24.24 | 23.9 | 23.99 | 23.36 | 23.89 | 24.71 | 24.37 | 24.29 |
| hsa-miR-26b*-4395555 | 35.42 | 33.98 | 33.47 | 33.69 | 32.6 | 33.35 | 33.96 | 35.9 | 35.32 |
| hsa-miR-26b-4395167 | 25.96 | 26.57 | 26.56 | 26.65 | 26.33 | 26.92 | 27.38 | 27.13 | 26.99 |
| hsa-miR-27a*-4395556 | 32.89 | 40 | 33.64 | 33.63 | 33.16 | 33.26 | 32.8 | 32.64 | 32.96 |
| hsa-miR-27a-4373287 | 28.97 | 30.19 | 29.79 | 29.81 | 29.19 | 30.21 | 28.42 | 29.64 | 28.95 |
| hsa-miR-27b*-4395285 | 31.29 | 31.54 | 30.98 | 32.25 | 33.97 | 30.81 | 30.59 | 32.61 | 31.11 |
| hsa-miR-27b-4373068 | 30.96 | 30.25 | 30.39 | 29.66 | 29.95 | 30.96 | 29.68 | 29.01 | 28.4 |
| hsa-miR-28-3p-4395557 | 29.59 | 29.9 | 29.98 | 29.97 | 29.74 | 29.62 | 30.87 | 30.54 | 30.53 |
| hsa-miR-28-5p-4373067 | 30.81 | 31.68 | 31.12 | 30.89 | 30.59 | 30.82 | 31.32 | 30.97 | 31.01 |
| hsa-miR-296-3p-4395212 | 30.65 | 30.77 | 30.51 | 32.19 | 30.92 | 31.06 | 36.01 | 40 | 40 |
| hsa-miR-296-5p-4373066 | 29.95 | 30.75 | 30.71 | 30.25 | 29.8 | 30.98 | 33.63 | 36.06 | 33.61 |
| hsa-miR-298-4395301 | 40 | 40 | 40 | 40 | 40 | 40 | 40 | 40 | 40 |
| hsa-miR-299-3p-4373189 | 33.83 | 40 | 35.25 | 35.08 | 34.23 | 31.32 | 40 | 33.6 | 31.09 |
| hsa-miR-299-5p-4373188 | 33.84 | 34.99 | 33.83 | 33.28 | 33.14 | 35 | 34.74 | 32.98 | 33.91 |
| hsa-miR-29a*-4395558 | 31.55 | 32.26 | 32.65 | 32.96 | 32.4 | 33.86 | 35.01 | 31.66 | 31.49 |
| hsa-miR-29a-4395223 | 24.4 | 25.3 | 25.39 | 25.14 | 24.93 | 25.53 | 24.19 | 22.88 | 22.36 |
| hsa-miR-29b-1*-4395276 | 40 | 40 | 40 | 40 | 40 | 40 | 40 | 35.99 | 40 |
| hsa-miR-29b-2*-4395277 | 35.9 | 40 | 40 | 31.41 | 33.23 | 32.23 | 29.49 | 29.75 | 28.91 |
| hsa-miR-29b-4373288 | 32.21 | 33.12 | 33.99 | 35.56 | 34.71 | 33.99 | 32.15 | 29.08 | 29.23 |
| hsa-miR-29c*-4381131 | 31.71 | 32.11 | 33.03 | 32.14 | 32.41 | 32 | 30.73 | 28.83 | 28.21 |
| hsa-miR-29c-4395171 | 30.36 | 31.23 | 31.17 | 30.81 | 30.98 | 31.39 | 30.03 | 28.36 | 27.98 |
| hsa-miR-301a-4373064 | 26.35 | 27.24 | 26.98 | 26.96 | 26.7 | 27.19 | 29.31 | 29.77 | 29.57 |
| hsa-miR-301b-4395503 | 27.85 | 29.35 | 28.97 | 29.48 | 28.8 | 29.28 | 32.84 | 33.43 | 33.32 |
| hsa-miR-302a*-4395492 | 31.58 | 31.71 | 31.37 | 31.71 | 31.01 | 31.09 | 30.72 | 31.67 | 31.18 |
| hsa-miR-302a-4378070 | 37.78 | 36.36 | 31.63 | 33.98 | 33.84 | 36.6 | 36.28 | 10.42 | 40 |
| hsa-miR-302b*-4395230 | 40 | 40 | 40 | 40 | 40 | 40 | 35.31 | 40 | 40 |
| hsa-miR-302b-4378071 | 40 | 33.92 | 40 | 40 | 40 | 37.12 | 35.25 | 40 | 33.35 |
| hsa-miR-302c*-4373277 | 40 | 32.13 | 32.29 | 32.54 | 34.53 | 32.96 | 32.74 | 32.39 | 31.6 |
| hsa-miR-302c-4378072 | 40 | 37.21 | 35.69 | 40 | 36.92 | 40 | 35.12 | 30.52 | 40 |
| hsa-miR-302d*-4395231 | 40 | 40 | 40 | 40 | 40 | 32.96 | 40 | 40 | 32.94 |
| hsa-miR-302d-4373063 | 31.15 | 30.43 | 30.58 | 32.03 | 32.45 | 31.11 | 40 | 34.17 | 31.88 |
| hsa-miR-30a*-4373062 | 26.93 | 26.65 | 26.85 | 26.93 | 26.55 | 27.16 | 26.67 | 26.67 | 26.05 |
| hsa-miR-30a-4373061 | 27.04 | 27.19 | 26.94 | 26.98 | 26.65 | 27.04 | 27.9 | 27.25 | 26.89 |
| hsa-miR-30b*-4395240 | 33.1 | 40 | 40 | 34.89 | 40 | 40 | 40 | 40 | 40 |
| hsa-miR-30b-4373290 | 25.97 | 26.07 | 25.76 | 25.66 | 25.47 | 25.78 | 26.15 | 26.14 | 25.33 |
| hsa-miR-30c-1*-4395219 | 29.95 | 28.94 | 29.37 | 31.42 | 29.63 | 30.73 | 31.54 | 40 | 31.8 |
| hsa-miR-30c-4373060 | 25.61 | 25.74 | 25.4 | 25.17 | 24.96 | 25.31 | 25.96 | 25.38 | 24.77 |
| hsa-miR-30d*-4395416 | 30.55 | 30.99 | 31.08 | 31.01 | 30.44 | 30.67 | 32.33 | 31.8 | 31.36 |
| hsa-miR-30d-4373059 | 26.76 | 26.97 | 28.27 | 26.82 | 26.46 | 26.88 | 27.66 | 27.15 | 26.74 |
| hsa-miR-30e*-4373057 | 26.74 | 26.93 | 26.67 | 26.52 | 26.57 | 26.9 | 26.94 | 26.73 | 26.38 |
| hsa-miR-30e-4395334 | 27.37 | 26.95 | 26.84 | 26.83 | 26.53 | 26.92 | 27.63 | 26.94 | 26.75 |
| hsa-miR-31-4395390 | 29.91 | 30.21 | 30.99 | 29.44 | 30.61 | 30.6 | 27.63 | 25.63 | 25.27 |
| hsa-miR-32*-4395222 | 40 | 40 | 40 | 40 | 40 | 29.31 | 40 | 40 | 35.02 |
| hsa-miR-320-4395388 | 24.33 | 24.59 | 24.67 | 24.48 | 24.36 | 24.49 | 26.79 | 26.93 | 26.96 |
| hsa-miR-323-3p-4395338 | 29.46 | 29.72 | 29.5 | 28.67 | 28.99 | 29.23 | 28.34 | 27.81 | 27.95 |
| hsa-miR-32-4395220 | 32.49 | 33.94 | 32.99 | 33.13 | 32.51 | 33.57 | 33.43 | 33.7 | 35.35 |
| hsa-miR-324-3p-4395272 | 28.69 | 28.89 | 28.97 | 28.87 | 28.78 | 28.49 | 29.57 | 29.22 | 29.04 |
| hsa-miR-324-5p-4373052 | 27.69 | 28.2 | 28.19 | 28.13 | 28 | 27.84 | 29.22 | 28.94 | 28.62 |
| hsa-miR-325-4373051 | 40 | 40 | 40 | 36 | 40 | 40 | 40 | 40 | 40 |
| hsa-miR-326-4373050 | 32.12 | 33.99 | 32.8 | 32.47 | 30.84 | 31.03 | 33.82 | 34.18 | 33.85 |
| hsa-miR-328-4373049 | 29.17 | 29.17 | 29.13 | 28.97 | 28.44 | 28.76 | 28.78 | 28.41 | 27.79 |
| hsa-miR-329-4373191 | 37.21 | 33.33 | 40 | 31.97 | 40 | 31.19 | 36.76 | 40 | 36.42 |
| hsa-miR-330-3p-4373047 | 31.07 | 31.98 | 31.75 | 31.69 | 30.9 | 30.55 | 29.75 | 28.94 | 28.61 |
| hsa-miR-330-5p-4395341 | 40 | 33.26 | 34.09 | 29.33 | 30.02 | 31.22 | 30.75 | 29.67 | 29.22 |
| hsa-miR-331-3p-4373046 | 24.49 | 24.92 | 24.76 | 24.61 | 24.19 | 24.48 | 25.98 | 26.07 | 25.38 |
| hsa-miR-331-5p-4395344 | 29.32 | 29.27 | 30.08 | 29.05 | 29.14 | 30.01 | 30.96 | 31.44 | 30.62 |
| hsa-miR-335*-4395296 | 29.96 | 30.11 | 29.89 | 29.82 | 29.96 | 30 | 30.8 | 30.36 | 30.98 |
| hsa-miR-335-4373045 | 29.97 | 29.47 | 29.34 | 29.04 | 29.33 | 29.78 | 29.45 | 29.77 | 29.24 |
| hsa-miR-337-3p-4395268 | 31.68 | 40 | 40 | 31.96 | 40 | 40 | 40 | 40 | 40 |
| hsa-miR-337-5p-4395267 | 31.2 | 33.34 | 33.99 | 31.75 | 32.46 | 32.09 | 32.11 | 32.43 | 32.03 |
| hsa-miR-338-3p-4395363 | 32.45 | 32.32 | 31.95 | 32 | 30.96 | 32.38 | 30.57 | 29.55 | 29.14 |
| hsa-miR-339-3p-4395295 | 29.66 | 29.31 | 29.32 | 29.13 | 28.66 | 28.98 | 30.75 | 29.96 | 29.95 |
| hsa-miR-339-5p-4395368 | 29.57 | 31.01 | 29.99 | 31.26 | 30.63 | 30.65 | 32.36 | 32.19 | 31.45 |
| hsa-miR-33a*-4395247 | 28.9 | 29.83 | 30 | 29.11 | 29.52 | 29.4 | 32.9 | 32.83 | 32.02 |
| hsa-miR-33b-4395196 | 28.76 | 40 | 30.01 | 28.34 | 29.86 | 29.93 | 40 | 27 | 40 |
| hsa-miR-340*-4395370 | 29.03 | 29.1 | 28.98 | 28.76 | 28.44 | 28.97 | 30.67 | 30.76 | 30.01 |
| hsa-miR-340-4395369 | 26.84 | 26.89 | 26.63 | 26.79 | 26.31 | 26.38 | 28.03 | 28.24 | 28.46 |
| hsa-miR-342-3p-4395371 | 24.39 | 24.16 | 23.97 | 24.05 | 22.99 | 23.55 | 24.85 | 24.45 | 24.83 |
| hsa-miR-342-5p-4395258 | 31.94 | 31.56 | 31.79 | 30.54 | 30.08 | 29.6 | 31.1 | 31.63 | 30.65 |
| hsa-miR-345-4395297 | 27.26 | 28.29 | 27.96 | 27.83 | 27.55 | 27.76 | 30.83 | 30.8 | 31.4 |
| hsa-miR-346-4373038 | 32.07 | 40 | 30.62 | 40 | 32.43 | 30.46 | 30.98 | 31.12 | 31.64 |
| hsa-miR-34a*-4395427 | 32.29 | 31.59 | 34.34 | 30.61 | 29.74 | 34.44 | 30.61 | 29.93 | 28.78 |
| hsa-miR-34a-4395168 | 30.98 | 30.95 | 31.38 | 30.93 | 30.46 | 31.85 | 28.86 | 26.98 | 26.37 |
| hsa-miR-34b*-4373037 | 40 | 31.06 | 30.69 | 30.52 | 30.41 | 31.69 | 34.96 | 40 | 40 |
| hsa-miR-34c-5p-4373036 | 34.24 | 34.62 | 32.53 | 32.18 | 32.28 | 32.98 | 33.92 | 35.97 | 40 |
| hsa-miR-361-3p-4395227 | 28.81 | 28.75 | 29.12 | 28.63 | 28.97 | 29.39 | 29.63 | 28.97 | 28.05 |
| hsa-miR-361-5p-4373035 | 30.51 | 31.02 | 30.14 | 30.29 | 29.75 | 30.13 | 30.83 | 30.84 | 30.58 |
| hsa-miR-362-3p-4395228 | 32.51 | 33.92 | 34.04 | 33.39 | 33.71 | 35.95 | 34.42 | 33.85 | 35.51 |
| hsa-miR-362-5p-4378092 | 30.72 | 31.54 | 31.6 | 31.45 | 31.18 | 32.45 | 31.96 | 31.75 | 32.98 |
| hsa-miR-363*-4380917 | 29.68 | 40 | 28.13 | 40 | 31.51 | 33.25 | 40 | 40 | 31.64 |
| hsa-miR-363-4378090 | 32.39 | 33.6 | 33.37 | 33.96 | 32.54 | 33.19 | 33.72 | 33.85 | 33.65 |
| hsa-miR-365-4373194 | 31.65 | 32.41 | 32.37 | 31.95 | 31.85 | 31.36 | 30.47 | 30.06 | 29.28 |
| hsa-miR-367*-4395232 | 40 | 40 | 32.21 | 40 | 40 | 40 | 31.55 | 40 | 40 |
| hsa-miR-367-4373034 | 40 | 40 | 40 | 40 | 34.77 | 40 | 40 | 40 | 40 |
| hsa-miR-369-3p-4373032 | 40 | 33.76 | 40 | 35.96 | 35.68 | 33.09 | 40 | 33.71 | 36.15 |
| hsa-miR-369-5p-4373195 | 32.27 | 32.47 | 32.49 | 31.97 | 36.99 | 31.29 | 33.35 | 33.28 | 34.96 |
| hsa-miR-370-4395386 | 29.19 | 28.99 | 28.79 | 28.36 | 29.61 | 28.69 | 28.22 | 27.74 | 27.53 |
| hsa-miR-371-3p-4395235 | 40 | 40 | 40 | 40 | 40 | 40 | 40 | 40 | 34.82 |
| hsa-miR-372-4373029 | 35.62 | 40 | 40 | 33.95 | 36.94 | 40 | 40 | 40 | 34.15 |
| hsa-miR-373*-4373279 | 28.07 | 28.31 | 28.99 | 29.89 | 29.16 | 28.88 | 40 | 40 | 40 |
| hsa-miR-373-4378073 | 36.98 | 36.05 | 34.02 | 36.09 | 35.3 | 36.06 | 40 | 40 | 40 |
| hsa-miR-374a*-4395236 | 34.96 | 34.39 | 33.33 | 33.01 | 32.63 | 32.2 | 40 | 36.96 | 32.81 |
| hsa-miR-374a-4373028 | 27.58 | 27.96 | 27.61 | 27.8 | 27.57 | 27.88 | 29.68 | 29.16 | 29.27 |
| hsa-miR-374b*-4395502 | 32.99 | 35.71 | 35.98 | 32.22 | 32.6 | 32.41 | 40 | 40 | 40 |
| hsa-miR-374b-4381045 | 25.83 | 25.79 | 25.32 | 25.48 | 25.38 | 25.66 | 26.97 | 27.52 | 27.64 |
| hsa-miR-375-4373027 | 40 | 33.5 | 35.89 | 33.25 | 40 | 40 | 36.95 | 40 | 34.03 |
| hsa-miR-376a*-4395238 | 33.31 | 32.63 | 32.99 | 33.4 | 35.95 | 33.85 | 36.13 | 32.51 | 33.04 |
| hsa-miR-376a-4373026 | 30.64 | 30.54 | 30.28 | 29.85 | 30.53 | 30.97 | 30.46 | 30.6 | 30.8 |
| hsa-miR-376b-4373196 | 40 | 40 | 40 | 40 | 40 | 40 | 40 | 40 | 40 |
| hsa-miR-376c-4395233 | 27.92 | 27.99 | 27.59 | 27.05 | 27.29 | 27.65 | 27.56 | 27.79 | 27.84 |
| hsa-miR-377*-4395239 | 31.75 | 33.91 | 31.16 | 30.7 | 33.9 | 30.68 | 32.05 | 32.16 | 30.7 |
| hsa-miR-377-4373025 | 40 | 40 | 33.89 | 40 | 40 | 40 | 40 | 40 | 36 |
| hsa-miR-378*-4373024 | 33.35 | 34.95 | 33.51 | 33.44 | 34.69 | 34.95 | 34.32 | 33.43 | 34.69 |
| hsa-miR-378-4395354 | 27.79 | 28.03 | 28.19 | 27.92 | 28.3 | 28.24 | 27.22 | 26.74 | 26.94 |
| hsa-miR-379*-4395244 | 40 | 40 | 40 | 34.27 | 35.03 | 34.55 | 40 | 35.76 | 40 |
| hsa-miR-379-4373349 | 29.95 | 29.79 | 29.91 | 28.98 | 29.7 | 29.5 | 28.18 | 27.98 | 27.42 |
| hsa-miR-380*-4373021 | 31.83 | 30.75 | 31.18 | 30.5 | 31.47 | 31.14 | 31.94 | 31.02 | 30.72 |
| hsa-miR-380-4373022 | 35.96 | 40 | 40 | 34.98 | 37.79 | 34.37 | 40 | 38.93 | 33.67 |
| hsa-miR-381-4373020 | 35.41 | 34.95 | 31.57 | 31.42 | 34.6 | 36.46 | 31.84 | 32.26 | 35.66 |
| hsa-miR-382-4373019 | 28.86 | 29.77 | 29.45 | 29 | 28.41 | 28.24 | 27.84 | 27.82 | 28.19 |
| hsa-miR-383-4373018 | 33.02 | 30.94 | 30.25 | 30.03 | 29.88 | 30.48 | 27.66 | 27.71 | 27.48 |
| hsa-miR-384-4373017 | 40 | 40 | 40 | 40 | 40 | 40 | 40 | 40 | 40 |
| hsa-miR-409-3p-4395443 | 29.45 | 30.61 | 31.13 | 29.96 | 30.85 | 30.3 | 30.5 | 29.61 | 29.35 |
| hsa-miR-409-5p-4395442 | 29.63 | 31.1 | 35.18 | 30.49 | 30.51 | 31.93 | 30.71 | 30.45 | 29.41 |
| hsa-miR-410-4378093 | 32.96 | 32.98 | 32.8 | 31.94 | 32.5 | 32.02 | 31.11 | 30.76 | 30.35 |
| hsa-miR-411*-4395349 | 40 | 40 | 32.9 | 32.22 | 36 | 32.39 | 33.56 | 35.2 | 31.21 |
| hsa-miR-411-4381013 | 29.83 | 29.58 | 29.9 | 28.59 | 28.72 | 29.31 | 28.47 | 28.36 | 27.97 |
| hsa-miR-412-4373199 | 40 | 31.87 | 31.29 | 33.3 | 32.1 | 34.44 | 32.78 | 33.48 | 31.95 |
| hsa-miR-422a-4395408 | 31.77 | 32.98 | 32.3 | 32.6 | 32.33 | 33.61 | 32.78 | 32.83 | 33.75 |
| hsa-miR-423-5p-4395451 | 27.25 | 28.79 | 28.72 | 27.75 | 27.36 | 28.26 | 28.97 | 29.72 | 29.97 |
| hsa-miR-424*-4395420 | 27.68 | 27.06 | 28.18 | 28.11 | 27.93 | 28.69 | 28.95 | 27.92 | 28.89 |
| hsa-miR-424-4373201 | 40 | 39.4 | 32.33 | 34.11 | 37.02 | 32.65 | 31.54 | 31.67 | 32.18 |
| hsa-miR-425*-4395413 | 31.6 | 32.39 | 32.96 | 32.3 | 32.13 | 32.56 | 31.58 | 32.03 | 32.02 |
| hsa-miR-425-4380926 | 26.97 | 27.6 | 27.3 | 27.71 | 27.34 | 27.26 | 27.98 | 27.18 | 27.09 |
| hsa-miR-429-4373203 | 36 | 33.78 | 31.86 | 31.49 | 32.9 | 32.14 | 40 | 33.25 | 33.54 |
| hsa-miR-431*-4395423 | 40 | 40 | 40 | 40 | 40 | 40 | 40 | 35.13 | 40 |
| hsa-miR-431-4395173 | 29.18 | 30.37 | 30.14 | 28.38 | 29.19 | 31.02 | 29.44 | 27.46 | 27.99 |
| hsa-miR-432*-4378076 | 31.44 | 31.59 | 31.89 | 30.84 | 32.02 | 32.04 | 30.94 | 32.08 | 30.66 |
| hsa-miR-432-4373280 | 30.08 | 30.78 | 31.28 | 29.89 | 31.61 | 30.72 | 29.77 | 29.11 | 29.35 |
| hsa-miR-433-4373205 | 30.65 | 30.11 | 30.16 | 29.36 | 30.91 | 31.01 | 28.63 | 27.71 | 27.97 |
| hsa-miR-448-4373206 | 40 | 40 | 32.51 | 32.06 | 33.99 | 40 | 35.99 | 32.98 | 40 |
| hsa-miR-449a-4373207 | 33.16 | 28.75 | 26.75 | 27.15 | 28.23 | 35.84 | 31.13 | 30.45 | 31.46 |
| hsa-miR-449b-4381011 | 31.96 | 29.75 | 28.05 | 28.12 | 28.63 | 31.33 | 31.29 | 31.89 | 30.97 |
| hsa-miR-450a-4395414 | 40 | 40 | 40 | 32.96 | 32.08 | 40 | 34.26 | 30.49 | 33.9 |
| hsa-miR-450b-3p-4395319 | 40 | 40 | 36.56 | 40 | 35.88 | 32.88 | 32.62 | 34.72 | 35.2 |
| hsa-miR-450b-5p-4395318 | 33.91 | 31.96 | 40 | 40 | 40 | 40 | 35.1 | 32.14 | 34.54 |
| hsa-miR-451-4373360 | 32.75 | 32.51 | 32.95 | 32.5 | 32.58 | 32.66 | 29.96 | 30.29 | 30.95 |
| hsa-miR-452*-4395441 | 40 | 33.93 | 40 | 40 | 40 | 33.97 | 40 | 40 | 40 |
| hsa-miR-452-4395440 | 29.79 | 30.87 | 30.9 | 31.29 | 32 | 31.34 | 33 | 32.73 | 31.45 |
| hsa-miR-453-4395429 | 40 | 40 | 40 | 36 | 40 | 40 | 40 | 40 | 40 |
| hsa-miR-454*-4395185 | 31.54 | 32.72 | 33.52 | 32.67 | 33.14 | 32.96 | 40 | 34.03 | 35.97 |
| hsa-miR-454-4395434 | 24.13 | 24.96 | 24.96 | 24.98 | 24.72 | 25.38 | 27.71 | 27.81 | 27.74 |
| hsa-miR-455-3p-4395355 | 29.3 | 31.59 | 30.39 | 30.52 | 31.21 | 31.45 | 31.85 | 36.05 | 32.94 |
| hsa-miR-455-5p-4378098 | 29.79 | 30.85 | 30.57 | 30.88 | 31.03 | 31.48 | 31.99 | 31.57 | 31.96 |
| hsa-miR-483-5p-4395449 | 30 | 30.63 | 31.85 | 30.21 | 30.88 | 29.81 | 30.35 | 31.97 | 31.07 |
| hsa-miR-484-4381032 | 23.37 | 23.95 | 23.48 | 23.7 | 22.92 | 23.53 | 25.46 | 25.46 | 25.6 |
| hsa-miR-485-3p-4378095 | 32.43 | 33.27 | 32.26 | 31.99 | 31.54 | 32.11 | 32.92 | 31.48 | 31.37 |
| hsa-miR-485-5p-4373212 | 29.01 | 28.92 | 31.89 | 28.45 | 40 | 40 | 28.13 | 28.19 | 28.03 |
| hsa-miR-486-3p-4395204 | 30.23 | 30.99 | 30.17 | 30.29 | 28.92 | 30.42 | 29.98 | 30.53 | 30.73 |
| hsa-miR-486-5p-4378096 | 29.46 | 29.96 | 29.33 | 29.68 | 29.43 | 29.15 | 29.31 | 28.9 | 29.77 |
| hsa-miR-487a-4378097 | 30.75 | 32.97 | 30.58 | 30.21 | 31.08 | 30.81 | 31.08 | 30.66 | 31.17 |
| hsa-miR-487b-4378102 | 30.32 | 30.96 | 30.61 | 30.23 | 30.16 | 30.52 | 29.47 | 28.92 | 28.82 |
| hsa-miR-488*-4373213 | 33.31 | 33.2 | 34.56 | 31.64 | 31.38 | 32.7 | 34.67 | 40 | 35.96 |
| hsa-miR-488-4395468 | 30.95 | 30.76 | 29.96 | 30.39 | 29.42 | 29.5 | 30.27 | 30.41 | 30.17 |
| hsa-miR-489-4395469 | 35.64 | 32.96 | 29.74 | 29.88 | 33.53 | 36.93 | 32.62 | 30.68 | 30.49 |
| hsa-miR-490-3p-4373215 | 32.01 | 29.87 | 30.45 | 29.96 | 30.61 | 30.65 | 28.52 | 29.3 | 28.91 |
| hsa-miR-491-3p-4395471 | 40 | 40 | 40 | 40 | 40 | 40 | 40 | 40 | 33.94 |
| hsa-miR-491-5p-4381053 | 30.58 | 29.8 | 29.97 | 29.37 | 29.62 | 29.63 | 29.76 | 28.66 | 27.87 |
| hsa-miR-492-4373217 | 40 | 40 | 40 | 40 | 40 | 40 | 40 | 40 | 40 |
| hsa-miR-493-4395475 | 31.47 | 30.35 | 33.34 | 31.83 | 35.93 | 36 | 32.01 | 33.11 | 36.96 |
| hsa-miR-494-4395476 | 28.44 | 29.11 | 29.91 | 28.98 | 29.51 | 28.26 | 28.22 | 28.69 | 28.16 |
| hsa-miR-495-4381078 | 30.45 | 31.3 | 31.42 | 30.83 | 30.86 | 30.94 | 30.11 | 29.13 | 29.06 |
| hsa-miR-496-4386771 | 40 | 40 | 40 | 32.01 | 35.87 | 32.82 | 32.04 | 40 | 36.03 |
| hsa-miR-497*-4395479 | 40 | 40 | 40 | 35.99 | 40 | 40 | 40 | 40 | 40 |
| hsa-miR-497-4373222 | 31.94 | 29.75 | 30.84 | 29.99 | 29.47 | 29.82 | 28.47 | 29.04 | 28.16 |
| hsa-miR-498-4373223 | 40 | 31.38 | 40 | 40 | 40 | 40 | 40 | 40 | 40 |
| hsa-miR-499-5p-4381047 | 33.41 | 40 | 40 | 32.82 | 29.56 | 40 | 29.18 | 31.13 | 29.57 |
| hsa-miR-500*-4373225 | 29 | 40 | 40 | 36 | 40 | 40 | 40 | 40 | 40 |
| hsa-miR-500-4395539 | 31.13 | 32.89 | 31.98 | 31.91 | 31.64 | 32.15 | 33.65 | 33.08 | 32.02 |
| hsa-miR-501-3p-4395546 | 40 | 32.1 | 31.36 | 40 | 40 | 32.39 | 40 | 40 | 40 |
| hsa-miR-501-5p-4373226 | 28.09 | 31.62 | 29.48 | 30.21 | 31.08 | 30.99 | 33 | 31.66 | 30.04 |
| hsa-miR-502-3p-4395194 | 30.81 | 32.76 | 32.67 | 36.31 | 31.99 | 33.98 | 40 | 31.9 | 33.76 |
| hsa-miR-502-5p-4373227 | 33.56 | 40 | 32.33 | 31.15 | 31.99 | 36.03 | 33.63 | 32.06 | 32.57 |
| hsa-miR-503-4373228 | 31 | 31.21 | 40 | 31.55 | 31.12 | 33.02 | 30.77 | 32.67 | 35.92 |
| hsa-miR-504-4395195 | 32.95 | 32.33 | 31.4 | 30.93 | 30.18 | 31.37 | 31.49 | 33.56 | 31.39 |
| hsa-miR-505*-4395198 | 29.06 | 29.47 | 29.67 | 29.4 | 29.41 | 29.68 | 31.59 | 32.98 | 31.35 |
| hsa-miR-505-4395200 | 28.23 | 29.82 | 29.68 | 29.02 | 28.74 | 28.46 | 29.95 | 30.22 | 40 |
| hsa-miR-506-4373231 | 33.05 | 40 | 40 | 40 | 40 | 40 | 40 | 40 | 40 |
| hsa-miR-508-3p-4373233 | 31.98 | 31.18 | 30.8 | 31.25 | 30.81 | 31.5 | 28.94 | 30.87 | 31.46 |
| hsa-miR-509-3-5p-4395266 | 40 | 40 | 40 | 40 | 33.94 | 40 | 40 | 40 | 40 |
| hsa-miR-509-3p-4395347 | 32.08 | 31.05 | 31.5 | 30.77 | 31.97 | 31.59 | 29.65 | 29.8 | 30.94 |
| hsa-miR-509-5p-4395346 | 32.7 | 32.49 | 30.89 | 31.26 | 33.97 | 31.72 | 30.46 | 30.3 | 32.27 |
| hsa-miR-510-4395352 | 40 | 40 | 40 | 40 | 40 | 40 | 34.05 | 40 | 40 |
| hsa-miR-511-4373236 | 33.05 | 33.99 | 35.52 | 34.42 | 35.96 | 33.65 | 33.22 | 31.98 | 32.08 |
| hsa-miR-512-3p-4381034 | 30.85 | 31.81 | 40 | 40 | 33.04 | 34.07 | 31.69 | 40 | 40 |
| hsa-miR-512-5p-4373238 | 40 | 40 | 40 | 40 | 40 | 40 | 36.69 | 40 | 40 |
| hsa-miR-513-3p-4395202 | 31.62 | 31.3 | 32.16 | 32.06 | 31.63 | 32.18 | 31.98 | 33.16 | 33.95 |
| hsa-miR-515-3p-4395480 | 40 | 40 | 35.61 | 35.48 | 40 | 40 | 40 | 37.13 | 35.05 |
| hsa-miR-515-5p-4373242 | 40 | 40 | 40 | 40 | 40 | 40 | 40 | 40 | 40 |
| hsa-miR-516a-3p-4373183 | 34.01 | 33.98 | 32.07 | 33.91 | 32.61 | 33.25 | 33.36 | 32.95 | 34.22 |
| hsa-miR-516a-5p-4395527 | 40 | 40 | 40 | 40 | 34 | 40 | 40 | 40 | 40 |
| hsa-miR-516b-4395172 | 40 | 40 | 40 | 40 | 40 | 40 | 40 | 36.18 | 40 |
| hsa-miR-517*-4378078 | 40 | 33.76 | 40 | 34.92 | 40 | 32.4 | 40 | 34.11 | 40 |
| hsa-miR-517a-4395513 | 32.18 | 31.42 | 34.75 | 33.4 | 31.24 | 32.17 | 30.07 | 29.28 | 29.13 |
| hsa-miR-517b-4373244 | 40 | 40 | 32.97 | 31.8 | 32.88 | 36.81 | 32.07 | 40 | 31.67 |
| hsa-miR-517c-4373264 | 36.02 | 33.46 | 35.64 | 36.23 | 33.96 | 33 | 34.33 | 33.2 | 30.53 |
| hsa-miR-518a-3p-4395508 | 31.66 | 40 | 40 | 40 | 40 | 32.16 | 40 | 40 | 40 |
| hsa-miR-518b-4373246 | 32.3 | 32.41 | 32.2 | 32.69 | 32.69 | 32.81 | 32.32 | 33.5 | 33.96 |
| hsa-miR-518c-4395512 | 40 | 40 | 36.04 | 40 | 40 | 40 | 40 | 40 | 40 |
| hsa-miR-518d-3p-4373248 | 32.03 | 40 | 29.66 | 31.43 | 31.06 | 29.79 | 29.58 | 29.45 | 30.23 |
| hsa-miR-518d-5p-4395500 | 33.23 | 40 | 40 | 40 | 32.61 | 40 | 40 | 40 | 40 |
| hsa-miR-518e-4395506 | 33.1 | 32.13 | 32.04 | 33.83 | 32.87 | 34.06 | 32.23 | 31.4 | 35.35 |
| hsa-miR-518f-4395499 | 35.01 | 35.56 | 34.31 | 36.54 | 35.93 | 36.83 | 35.52 | 38.7 | 32.76 |
| hsa-miR-519a-4395526 | 31.98 | 33.15 | 32.69 | 32.14 | 32.02 | 33.04 | 36.03 | 31.44 | 31.45 |
| hsa-miR-519b-3p-4395495 | 33.56 | 33.39 | 33.03 | 34.3 | 33.8 | 34.95 | 34.45 | 33.2 | 33.65 |
| hsa-miR-519c-3p-4373251 | 35.76 | 40 | 32.79 | 40 | 40 | 40 | 40 | 40 | 40 |
| hsa-miR-519d-4395514 | 34.05 | 31.26 | 32.01 | 33.64 | 40 | 30.94 | 31.45 | 31.56 | 31.99 |
| hsa-miR-519e-4395481 | 33.08 | 35.5 | 40 | 33.69 | 32.82 | 40 | 33.1 | 40 | 33.35 |
| hsa-miR-520a-3p-4373268 | 40 | 35.38 | 40 | 40 | 33.87 | 40 | 33.07 | 32.77 | 40 |
| hsa-miR-520b-4373252 | 40 | 40 | 35.85 | 37.44 | 40 | 40 | 40 | 40 | 40 |
| hsa-miR-520c-3p-4395511 | 34.96 | 33.28 | 33.34 | 34.83 | 35.57 | 34.8 | 33.84 | 34.99 | 34.86 |
| hsa-miR-520d-5p-4395504 | 40 | 40 | 40 | 40 | 40 | 33.84 | 40 | 40 | 40 |
| hsa-miR-520f-4373256 | 33.24 | 40 | 34.79 | 40 | 36.03 | 38.38 | 40 | 34.27 | 40 |
| hsa-miR-520g-4373257 | 39.3 | 40 | 40 | 40 | 40 | 40 | 34.72 | 36.1 | 33.72 |
| hsa-miR-520h-4373258 | 34.09 | 30.57 | 32.11 | 31.8 | 32.8 | 33.78 | 40 | 33.14 | 30.81 |
| hsa-miR-521-4373259 | 40 | 40 | 40 | 40 | 34.19 | 33.46 | 40 | 33.23 | 40 |
| hsa-miR-522-4395524 | 33.95 | 31.51 | 32.02 | 31.93 | 30.01 | 30.89 | 31.3 | 32 | 33.06 |
| hsa-miR-523-4395497 | 32.67 | 32.79 | 35.49 | 33.43 | 34.87 | 32.93 | 35.02 | 34.9 | 37.05 |
| hsa-miR-524-3p-4378087 | 32.7 | 32.26 | 32.61 | 33.28 | 31.92 | 32.81 | 32.63 | 31.07 | 31.38 |
| hsa-miR-525-3p-4395496 | 40 | 40 | 40 | 40 | 40 | 40 | 40 | 40 | 35.96 |
| hsa-miR-525-5p-4378088 | 40 | 40 | 40 | 40 | 32.87 | 40 | 40 | 40 | 40 |
| hsa-miR-526b*-4395494 | 32.82 | 32.88 | 32.33 | 32.95 | 33.31 | 35 | 32.74 | 34.32 | 33.5 |
| hsa-miR-532-3p-4395466 | 28.94 | 30.95 | 30.53 | 30.37 | 30.75 | 31.07 | 31.94 | 31.83 | 32.01 |
| hsa-miR-532-5p-4380928 | 27.68 | 28.99 | 28.98 | 28.82 | 28.65 | 29.9 | 30.66 | 30.01 | 30.42 |
| hsa-miR-539-4378103 | 30.94 | 30.51 | 30.48 | 30.08 | 29.99 | 31.21 | 29.6 | 28.38 | 27.63 |
| hsa-miR-541*-4395311 | 40 | 40 | 40 | 34.57 | 40 | 40 | 40 | 36.77 | 35.97 |
| hsa-miR-541-4395312 | 34.05 | 32.55 | 40 | 40 | 31.49 | 40 | 35.83 | 31.96 | 31.94 |
| hsa-miR-542-3p-4378101 | 31.47 | 34.9 | 33.76 | 40 | 31.26 | 40 | 30.82 | 40 | 32.69 |
| hsa-miR-542-5p-4395351 | 31.05 | 40 | 32.97 | 30.89 | 31.63 | 40 | 29.63 | 32.43 | 33.94 |
| hsa-miR-543-4395487 | 32.97 | 32.8 | 33.43 | 31.75 | 32.7 | 33.35 | 32.02 | 31.71 | 31.23 |
| hsa-miR-544-4395376 | 40 | 40 | 40 | 35.5 | 40 | 36.98 | 35.6 | 40 | 40 |
| hsa-miR-545*-4395377 | 31.69 | 32.1 | 33.38 | 34.26 | 32.42 | 31.8 | 34.9 | 34.71 | 35.32 |
| hsa-miR-545-4395378 | 31.13 | 32.33 | 31.08 | 31.77 | 30.42 | 31.82 | 32.03 | 40 | 35.47 |
| hsa-miR-548a-3p-4380948 | 40 | 40 | 40 | 40 | 35.83 | 40 | 40 | 40 | 40 |
| hsa-miR-548b-5p-4395519 | 30.64 | 32.89 | 34.94 | 31.19 | 31.11 | 31.07 | 33.1 | 31.95 | 33.39 |
| hsa-miR-548c-5p-4395540 | 32.49 | 31.74 | 32.46 | 34.16 | 31.98 | 32.05 | 32.24 | 34.88 | 32.49 |
| hsa-miR-548d-3p-4381008 | 35.65 | 33.19 | 40 | 40 | 40 | 34.42 | 35.98 | 40 | 40 |
| hsa-miR-548d-5p-4395348 | 32.88 | 33.07 | 32.35 | 33.63 | 32.54 | 31.79 | 33.28 | 35.74 | 34.66 |
| hsa-miR-549-4380921 | 32.46 | 40 | 40 | 40 | 34.04 | 40 | 40 | 40 | 40 |
| hsa-miR-550*-4380954 | 30.33 | 30.9 | 29.99 | 29.95 | 29.86 | 29.89 | 31.95 | 31.89 | 32.97 |
| hsa-miR-550-4395521 | 32.39 | 32.68 | 33.49 | 32.41 | 32.67 | 32.35 | 33.3 | 33.97 | 34.11 |
| hsa-miR-551a-4380929 | 32.21 | 32.05 | 34.28 | 40 | 40 | 40 | 40 | 40 | 33.21 |
| hsa-miR-551b*-4395457 | 32 | 33 | 32.52 | 32.61 | 31.52 | 31.84 | 33.86 | 32.87 | 33.05 |
| hsa-miR-551b-4380945 | 32.58 | 34.85 | 35.56 | 33.96 | 33.77 | 32.51 | 35.26 | 34.89 | 34.27 |
| hsa-miR-552-4380930 | 32.35 | 30.91 | 40 | 34.8 | 32.48 | 31.71 | 40 | 31.55 | 31.1 |
| hsa-miR-553-4380931 | 40 | 40 | 40 | 40 | 40 | 40 | 40 | 29.18 | 40 |
| hsa-miR-554-4380932 | 40 | 33.67 | 33.99 | 40 | 40 | 32.91 | 40 | 40 | 40 |
| hsa-miR-555-4380933 | 32.54 | 30.1 | 31.36 | 34.45 | 30.95 | 30.59 | 40 | 40 | 31.43 |
| hsa-miR-558-4380936 | 40 | 30.05 | 40 | 40 | 40 | 40 | 40 | 40 | 40 |
| hsa-miR-559-4380937 | 34.22 | 32.02 | 33.46 | 33.74 | 34.11 | 33.12 | 34.65 | 33.75 | 34.43 |
| hsa-miR-561-4380938 | 32.61 | 32.54 | 40 | 36.27 | 35.96 | 35.79 | 35.47 | 34.82 | 40 |
| hsa-miR-562-4380939 | 35.49 | 40 | 34.11 | 40 | 40 | 34.96 | 40 | 40 | 34.93 |
| hsa-miR-563-4380940 | 40 | 31.7 | 34.51 | 37.17 | 34.01 | 40 | 40 | 40 | 40 |
| hsa-miR-564-4380941 | 30.03 | 29.14 | 29.61 | 30.99 | 30.22 | 29.88 | 29.65 | 29.67 | 30.26 |
| hsa-miR-565-4380942 | 29.07 | 28.35 | 28.01 | 27.33 | 27.53 | 26.95 | 31.02 | 29.08 | 28.2 |
| hsa-miR-566-4380943 | 28.49 | 27.25 | 27.41 | 28.18 | 28.43 | 27.95 | 28.99 | 28.94 | 30.33 |
| hsa-miR-567-4380944 | 28.31 | 28.08 | 28.48 | 29.94 | 30.92 | 29.11 | 28.91 | 29.75 | 29.35 |
| hsa-miR-569-4380946 | 33.91 | 31.53 | 32.97 | 31.62 | 40 | 32.33 | 34.51 | 35.21 | 36.75 |
| hsa-miR-570-4395458 | 40 | 34.17 | 34.95 | 40 | 40 | 33.72 | 34.93 | 40 | 34.34 |
| hsa-miR-571-4381016 | 29.61 | 28.78 | 29.2 | 29.17 | 29.15 | 28.81 | 28.68 | 29.01 | 29.1 |
| hsa-miR-572-4381017 | 32.06 | 31.74 | 31.03 | 31.78 | 31.89 | 31.48 | 31.81 | 32.57 | 32.48 |
| hsa-miR-573-4381018 | 31.63 | 32.17 | 32.2 | 31.42 | 32.56 | 32.19 | 32.47 | 33.01 | 33.18 |
| hsa-miR-574-3p-4395460 | 27.54 | 28.5 | 28.1 | 28.11 | 27.87 | 28.23 | 29.98 | 28.94 | 29.41 |
| hsa-miR-575-4381020 | 30.65 | 29.76 | 29.44 | 29.98 | 30.73 | 30.03 | 31.13 | 31.19 | 32.09 |
| hsa-miR-576-3p-4395462 | 30 | 29.81 | 29.95 | 30.77 | 30.56 | 30.59 | 32.06 | 31.98 | 33.04 |
| hsa-miR-576-5p-4395461 | 37.22 | 40 | 40 | 33.95 | 35.05 | 40 | 40 | 40 | 40 |
| hsa-miR-578-4381022 | 30.72 | 33.65 | 32.66 | 31.7 | 36.16 | 40 | 31.89 | 40 | 40 |
| hsa-miR-579-4395509 | 33.16 | 34.6 | 33.24 | 33.95 | 32.37 | 32.8 | 35.41 | 34.47 | 35.05 |
| hsa-miR-580-4381024 | 34.31 | 33.11 | 34.17 | 33.57 | 32.99 | 32.56 | 33.67 | 34.13 | 33.85 |
| hsa-miR-581-4386744 | 34.93 | 32.12 | 33.61 | 33.59 | 34.38 | 40 | 34.59 | 36.28 | 34.09 |
| hsa-miR-582-3p-4395510 | 31.5 | 33.89 | 30.91 | 30.27 | 30.59 | 29.43 | 31.93 | 31.72 | 36.98 |
| hsa-miR-582-5p-4395175 | 32.97 | 30.93 | 31.8 | 30.97 | 30.84 | 30.52 | 33.21 | 33.59 | 33.57 |
| hsa-miR-583-4381025 | 30.77 | 29.77 | 30.46 | 30.38 | 30.47 | 30.6 | 30.87 | 30.89 | 31.28 |
| hsa-miR-584-4381026 | 30.1 | 29.26 | 30.02 | 29.48 | 30.09 | 30.42 | 28.8 | 28.36 | 28.34 |
| hsa-miR-585-4381027 | 40 | 40 | 40 | 30.28 | 29.17 | 40 | 40 | 40 | 40 |
| hsa-miR-586-4380949 | 35.2 | 32.62 | 33.76 | 33.93 | 34.13 | 32.36 | 34.74 | 36.1 | 33.86 |
| hsa-miR-587-4380950 | 31.71 | 30.68 | 31.6 | 32.03 | 31.99 | 31.53 | 31.54 | 31.33 | 31.55 |
| hsa-miR-589*-4380953 | 30.52 | 30.44 | 30.08 | 31.11 | 32.85 | 32.65 | 33.11 | 33.96 | 30.18 |
| hsa-miR-589-4395520 | 37 | 33.71 | 40 | 33.56 | 37.45 | 34.25 | 36.86 | 40 | 35.87 |
| hsa-miR-590-5p-4395176 | 28.34 | 28.47 | 28.03 | 28.09 | 28.02 | 28.75 | 30.48 | 30.94 | 30.63 |
| hsa-miR-591-4380955 | 30.06 | 29.96 | 31.14 | 30.6 | 30.48 | 31.28 | 31.36 | 31.33 | 30.1 |
| hsa-miR-592-4380956 | 30.5 | 30.21 | 29.96 | 29.38 | 29.8 | 30.08 | 28.77 | 28.73 | 28.12 |
| hsa-miR-593*-4380957 | 40 | 40 | 40 | 40 | 31.25 | 40 | 30.79 | 40 | 40 |
| hsa-miR-593-4395522 | 40 | 40 | 40 | 40 | 40 | 40 | 40 | 40 | 40 |
| hsa-miR-595-4395178 | 30.55 | 30.92 | 31.53 | 31.36 | 30.25 | 30.21 | 40 | 32.84 | 31.1 |
| hsa-miR-596-4380959 | 26 | 25.24 | 25.72 | 25.93 | 25.71 | 25.64 | 25.67 | 25.91 | 25.75 |
| hsa-miR-597-4380960 | 32.62 | 33.82 | 32.04 | 32.96 | 32.72 | 32.54 | 33.91 | 35.3 | 34.36 |
| hsa-miR-598-4395179 | 27.96 | 28.26 | 28.18 | 27.93 | 28 | 28.25 | 27.96 | 27.67 | 27.22 |
| hsa-miR-599-4380962 | 40 | 40 | 33.76 | 40 | 40 | 40 | 40 | 36.47 | 40 |
| hsa-miR-600-4380963 | 31.61 | 30.15 | 30.81 | 29.98 | 30.51 | 29.85 | 30.45 | 30.88 | 29.58 |
| hsa-miR-601-4380965 | 34.85 | 34.52 | 34.46 | 34.96 | 34.85 | 34.04 | 34.16 | 34.54 | 35.94 |
| hsa-miR-603-4380972 | 40 | 31.97 | 40 | 40 | 40 | Failed | 40 | 40 | 40 |
| hsa-miR-604-4380973 | 29.45 | 29.36 | 29.79 | 30.64 | 29.94 | 29.97 | 30.34 | 29.85 | 30.06 |
| hsa-miR-605-4386742 | 30.44 | 29.49 | 29.99 | 30.75 | 29.25 | 31.09 | 32.53 | 30.72 | 30.34 |
| hsa-miR-606-4380974 | 34.63 | 32.62 | 34.44 | 34.03 | 32.3 | 33.66 | 35.51 | 33.25 | 36.01 |
| hsa-miR-607-4380975 | 40 | 32.4 | 40 | 40 | 40 | 30.47 | 40 | 40 | 40 |
| hsa-miR-608-4380976 | 40 | 40 | 40 | 32.5 | 40 | 40 | 40 | 40 | 40 |
| hsa-miR-609-4380978 | 40 | 33.12 | 33.8 | 33.17 | 33.3 | 40 | 40 | 40 | 40 |
| hsa-miR-610-4380980 | 30.71 | 30.95 | 30.17 | 30.3 | 31.37 | 29.76 | 29.78 | 30.66 | 30.95 |
| hsa-miR-612-4380983 | 40 | 40 | 40 | 40 | 40 | Failed | Failed | 40 | 40 |
| hsa-miR-613-4380989 | 33.83 | 33.07 | 36.08 | 40 | 32.89 | 34 | 40 | 40 | 40 |
| hsa-miR-614-4380990 | 40 | 33.47 | 34.06 | 33.11 | 33.18 | 33.35 | 34.19 | 32.55 | 34.87 |
| hsa-miR-615-3p-4386777 | 40 | 40 | 28.46 | 40 | 40 | 40 | 40 | 40 | 40 |
| hsa-miR-616*-4380992 | 40 | 35.09 | 34.69 | 40 | Failed | 40 | 40 | 35.52 | 38.85 |
| hsa-miR-616-4395525 | 34.09 | 40 | 35.73 | 40 | 40 | 35.66 | 40 | 40 | 40 |
| hsa-miR-617-4380994 | 33.1 | 30.55 | 32.18 | 31.04 | 30.96 | 30.99 | 31.29 | 33.66 | 32.24 |
| hsa-miR-618-4380996 | 33.73 | 32.76 | 33.53 | 35 | 32.73 | 32.42 | 32.96 | 33.46 | 34 |
| hsa-miR-619-4380998 | 40 | 40 | 40 | 40 | 33.92 | 40 | 40 | 40 | 40 |
| hsa-miR-621-4381001 | 30.41 | 31.8 | 40 | 31.45 | 32.39 | 31.03 | 32.11 | 34.05 | 40 |
| hsa-miR-622-4380961 | 35.21 | 32.22 | 31.22 | 33.15 | 32.93 | 31.96 | 33.98 | 32.68 | 31.38 |
| hsa-miR-623-4386740 | 32.53 | 31.91 | 31.89 | 33.1 | 32.82 | 32.14 | 32.36 | 32.05 | 33.16 |
| hsa-miR-624*-4380964 | 36.69 | 36.44 | 40 | 36.74 | 40 | 32.63 | 37.14 | 40 | 40 |
| hsa-miR-624-4395541 | 40 | 40 | 40 | 40 | 36.62 | 40 | 40 | 40 | 40 |
| hsa-miR-625*-4395543 | 28.35 | 28.01 | 28.13 | 28.54 | 28.39 | 28.24 | 29.78 | 29.5 | 29.97 |
| hsa-miR-625-4395542 | 31.96 | 31.96 | 31.77 | 31.56 | 31.26 | 31.68 | 34.09 | 33.26 | 33.25 |
| hsa-miR-626-4380966 | 30.82 | 40 | 40 | 40 | 40 | 31.48 | 40 | 31.38 | 34.79 |
| hsa-miR-627-4380967 | 32.13 | 33.78 | 32.43 | 32.49 | 31.76 | 31.8 | 34.99 | 37.04 | 34.84 |
| hsa-miR-628-3p-4395545 | 31.45 | 30.94 | 31.05 | 30.82 | 30.7 | 30.75 | 30.06 | 29.46 | 29.35 |
| hsa-miR-628-5p-4395544 | 29.08 | 28.94 | 28.95 | 28.58 | 28.27 | 28.85 | 28.35 | 28.21 | 28.13 |
| hsa-miR-629*-4380969 | 31.6 | 31.24 | 30.58 | 30.8 | 31.12 | 31.33 | 34.33 | 34.54 | 33.96 |
| hsa-miR-629-4395547 | 33.29 | 31.11 | 31 | 30.94 | 31.8 | 30.92 | 32.35 | 32.88 | 35.93 |
| hsa-miR-630-4380970 | 29.77 | 29.7 | 30.52 | 30.48 | 29.66 | 30.52 | 31.45 | 31.39 | 31.52 |
| hsa-miR-631-4380971 | 40 | 26.49 | 29.84 | 40 | 40 | 40 | 40 | 28.84 | 40 |
| hsa-miR-632-4380977 | 29.36 | 30.64 | 30.39 | 30.68 | 30.5 | 30.46 | 30.99 | 32.48 | 31.04 |
| hsa-miR-633-4380979 | 40 | 26.74 | 40 | 32.01 | 25.9 | 40 | 40 | 40 | 40 |
| hsa-miR-634-4380981 | 40 | 40 | 40 | 40 | 40 | 40 | 30.77 | 40 | 30.75 |
| hsa-miR-635-4380982 | 32.18 | 34.93 | 32.49 | 32.52 | 35.4 | 33.29 | 31.85 | 40 | 36.9 |
| hsa-miR-636-4395199 | 33.95 | 32.98 | 33.16 | 32.88 | 32.94 | 32.24 | 32.53 | 34.04 | 33.63 |
| hsa-miR-637-4380985 | 40 | 31.69 | 40 | 33.68 | 22.96 | 40 | 40 | 40 | 40 |
| hsa-miR-638-4380986 | 31.88 | 29.79 | 30.34 | 30.77 | 30.83 | 30.37 | 30.98 | 30.21 | 30.8 |
| hsa-miR-639-4380987 | 29.31 | 28.99 | 29.01 | 29.52 | 30.18 | 29.99 | 30.27 | 29.93 | 29.4 |
| hsa-miR-640-4386743 | 32.28 | 30.01 | 31.55 | 31.63 | 31.95 | 32.24 | 31.47 | 31.63 | 31.53 |
| hsa-miR-641-4380988 | 32.49 | 31.31 | 31.47 | 31.19 | 32.85 | 32.97 | 34.11 | 40 | 40 |
| hsa-miR-642-4380995 | 37.01 | 33.56 | 40 | 30.94 | 31.62 | 32.14 | 40 | 32.08 | 35.73 |
| hsa-miR-643-4380997 | 30.75 | 29.35 | 30.12 | 31.05 | 32.01 | 30.68 | 30.79 | 30.95 | 31.32 |
| hsa-miR-644-4380999 | 29.88 | 29.34 | 29.44 | 29.7 | 30.27 | 29.57 | 29.97 | 29.95 | 30.22 |
| hsa-miR-645-4381000 | 40 | 40 | 37.43 | 33.85 | 40 | 35.5 | 36.83 | 35.96 | 33.75 |
| hsa-miR-646-4381002 | 29.68 | 29.32 | 29.65 | 29.04 | 30.71 | 30.45 | 31.99 | 30.84 | 31.3 |
| hsa-miR-648-4381004 | 31.02 | 30.61 | 31 | 31.03 | 31.3 | 31.32 | 31.94 | 31.94 | 31.59 |
| hsa-miR-649-4381005 | 32.31 | 30.8 | 31.48 | 31.49 | 32.23 | 31.58 | 33.13 | 31.97 | 32.13 |
| hsa-miR-650-4381006 | 27.09 | 27.18 | 27.62 | 27.78 | 27.3 | 27.53 | 28.49 | 27.96 | 28.35 |
| hsa-miR-651-4381007 | 40 | 34.83 | 40 | 40 | 40 | 40 | 40 | 40 | 35.88 |
| hsa-miR-652-4395463 | 29.74 | 30.38 | 29.99 | 29.81 | 30.23 | 29.84 | 30.64 | 29.93 | 29.93 |
| hsa-miR-654-3p-4395350 | 30.33 | 33.96 | 32 | 37.19 | 31.72 | 35.03 | 35.86 | 32.43 | 40 |
| hsa-miR-654-5p-4381014 | 29.34 | 30.04 | 29.51 | 29.28 | 29.95 | 32.82 | 29.84 | 28.83 | 29.44 |
| hsa-miR-655-4381015 | 32.53 | 32.65 | 31.95 | 31.58 | 31.81 | 32.15 | 31.94 | 31.81 | 30.82 |
| hsa-miR-656-4380920 | 33.62 | 32.7 | 33.63 | 33.07 | 32.32 | 32.69 | 31.73 | 31.32 | 29.94 |
| hsa-miR-657-4380922 | 28.84 | 28.95 | 28.14 | 29.3 | 29.37 | 29.64 | 29.36 | 29.59 | 29.49 |
| hsa-miR-658-4380923 | 40 | 31.19 | 40 | 40 | 40 | 40 | 40 | 40 | 32.73 |
| hsa-miR-659-4380924 | 29.96 | 29.25 | 30.19 | 30.52 | 30.86 | 30.16 | 30.98 | 31.5 | 32.32 |
| hsa-miR-660-4380925 | 27.48 | 28.45 | 28.43 | 28.42 | 28.41 | 28.78 | 28.88 | 28.92 | 28.89 |
| hsa-miR-661-4381009 | 28.09 | 27.19 | 27.29 | 28.1 | 28.13 | 27.71 | 28 | 27.56 | 28.78 |
| hsa-miR-662-4381010 | 30.26 | 30.13 | 40 | 30.73 | 32.73 | 30.9 | 30.03 | 40 | 40 |
| hsa-miR-668-4395181 | 30.32 | 31.02 | 31.37 | 30 | 31.78 | 33.27 | 29.73 | 28.51 | 28.27 |
| hsa-miR-671-3p-4395433 | 31.96 | 33.42 | 31.9 | 31.99 | 31.11 | 31.96 | 40 | 33.98 | 32.5 |
| hsa-miR-672-4395438 | 30.36 | 31.83 | 30.55 | 32.26 | 31.76 | 32.62 | 32.99 | 40 | 34.58 |
| hsa-miR-674-4395193 | 40 | 40 | Failed | 40 | 40 | 40 | 40 | 31.42 | 40 |
| hsa-miR-675-4395192 | 28.22 | 27.95 | 28.2 | 28.57 | 29.02 | 28.72 | 28.4 | 28.58 | 28.49 |
| hsa-miR-708*-4395453 | 34.03 | 30.95 | 31.42 | 33 | 32.72 | 40 | 31.91 | 40 | 31.68 |
| hsa-miR-708-4395452 | 25.95 | 26.03 | 25.89 | 25.93 | 25.61 | 25.62 | 27.3 | 27.62 | 26.98 |
| hsa-miR-7-1*-4381118 | 27.84 | 28.16 | 27.98 | 27.97 | 27.3 | 27.69 | 29.68 | 28.67 | 28.55 |
| hsa-miR-7-2*-4395425 | 33.74 | 32.77 | 33.99 | 31.93 | 32.53 | 33.47 | 34.9 | 32.85 | 31.07 |
| hsa-miR-7-4378130 | 32.18 | 30.6 | 29.31 | 29.66 | 31.3 | 31.73 | 28.59 | 27.34 | 27.92 |
| hsa-miR-744*-4395436 | 31.46 | 32.05 | 31.44 | 31.35 | 31.03 | 31.34 | 32.97 | 31.5 | 31.21 |
| hsa-miR-744-4395435 | 27.57 | 28.33 | 28.21 | 28.24 | 28.03 | 28.06 | 28.95 | 28.17 | 27.83 |
| hsa-miR-758-4395180 | 31.71 | 32.89 | 32.34 | 31.82 | 32.96 | 31.65 | 31.37 | 30.61 | 30.58 |
| hsa-miR-760-4395439 | 28.61 | 28.79 | 28.89 | 28.64 | 28.51 | 28.79 | 28.95 | 28.94 | 28.87 |
| hsa-miR-766-4395177 | 28.13 | 27.35 | 27.22 | 26.87 | 26.54 | 26.94 | 27.4 | 27.65 | 27.22 |
| hsa-miR-767-3p-4395184 | 36.17 | 31.62 | 40 | 34.48 | 40 | 40 | 40 | 40 | 32.77 |
| hsa-miR-767-5p-4395182 | 28.53 | 29.29 | 29.12 | 28.86 | 29.38 | 29.47 | 31.77 | 32.78 | 31.79 |
| hsa-miR-768-3p-4395188 | 26.56 | 25.47 | 25.42 | 25.14 | 24.85 | 24.82 | 26.08 | 26.32 | 25.86 |
| hsa-miR-769-3p-4395190 | 40 | 31.3 | 40 | 31.6 | 29.98 | 31.73 | 30.99 | 32.08 | 29.24 |
| hsa-miR-769-5p-4395186 | 28.84 | 28.64 | 28.58 | 28.84 | 28.68 | 28.73 | 27.7 | 27.09 | 27.31 |
| hsa-miR-770-5p-4395189 | 31.97 | 30.49 | 31.04 | 30.23 | 30.82 | 30.7 | 30.07 | 28.92 | 28.91 |
| hsa-miR-801-4395183 | 20.33 | 20.39 | 20.72 | 20.87 | 20.87 | 21.22 | 22.37 | 23.25 | 23.27 |
| hsa-miR-871-4395465 | 40 | 36.09 | 40 | 40 | 40 | 40 | 40 | 40 | 40 |
| hsa-miR-872-4395375 | 36.62 | 40 | 40 | 40 | 34.7 | 40 | 40 | 40 | 37.38 |
| hsa-miR-873-4395467 | 34.98 | 33.19 | 31.77 | 31.18 | 32.62 | 32.84 | 31.23 | 31.92 | 31.5 |
| hsa-miR-874-4395379 | 28.95 | 28.9 | 28.39 | 28.88 | 28.07 | 27.93 | 27.54 | 26.92 | 26.71 |
| hsa-miR-875-3p-4395315 | 34.35 | 32 | 40 | 40 | 40 | 40 | 40 | 40 | 40 |
| hsa-miR-875-5p-4395314 | 33.56 | 32.04 | 32.33 | 32.64 | 33.39 | 32.98 | 32.7 | 32.63 | 32.79 |
| hsa-miR-876-3p-4395336 | 32.63 | 34.01 | 36.03 | 30.91 | 32.36 | 31.83 | 32.29 | 32.67 | 31.08 |
| hsa-miR-876-5p-4395316 | 32.92 | 32.41 | 31.11 | 31.16 | 31.73 | 32.24 | 30.18 | 31.19 | 30.64 |
| hsa-miR-877-4395402 | 29.25 | 28.63 | 29.21 | 29.97 | 29.82 | 29.41 | 29.61 | 29.15 | 29.65 |
| hsa-miR-885-3p-4395483 | 29.68 | 40 | 29.92 | 40 | 28.62 | 40 | 28.74 | 28.26 | 25.68 |
| hsa-miR-885-5p-4395407 | 27.97 | 28.46 | 27.82 | 27.83 | 27.7 | 27.96 | 26.77 | 26.44 | 25.42 |
| hsa-miR-886-3p-4395305 | 26.88 | 27.18 | 27.33 | 26.82 | 26.81 | 27.33 | 29.44 | 28.96 | 28.19 |
| hsa-miR-886-5p-4395304 | 27.41 | 27.99 | 28 | 27.64 | 27.31 | 27.97 | 31.28 | 30.24 | 29.95 |
| hsa-miR-887-4395485 | 28.41 | 30.45 | 29.33 | 29.89 | 29.56 | 31.2 | 40 | 29.64 | 30.53 |
| hsa-miR-888-4395323 | 31.63 | 33.33 | 30.92 | 31.81 | 30.05 | 30.02 | 30.92 | 32.8 | 35.95 |
| hsa-miR-889-4395313 | 33.69 | 33.95 | 35.38 | 32.32 | 32.96 | 34.16 | 32.24 | 31.56 | 30.94 |
| hsa-miR-891a-4395302 | 34.23 | 32.28 | 30.38 | 31.64 | 31.94 | 30.64 | 35.73 | 36.87 | 34.08 |
| hsa-miR-892b-4395325 | 33.05 | 31.4 | 30.59 | 31.65 | 31.97 | 31.31 | 32.98 | 33.71 | 31.34 |
| hsa-miR-9*-4395342 | 22.64 | 22.8 | 22.54 | 22.95 | 22.83 | 22.9 | 23.4 | 23.63 | 23.37 |
| hsa-miR-920-4395261 | 40 | 31.12 | 40 | 40 | 40 | 40 | 40 | 40 | 40 |
| hsa-miR-921-4395262 | 31.33 | 31.05 | 40 | 33.04 | 34.01 | 32.35 | 32.7 | 31.82 | 40 |
| hsa-miR-922-4395263 | 33.47 | 31.75 | 31.32 | 33.02 | 31.62 | 32.04 | 33.5 | 34.04 | 31.75 |
| hsa-miR-923-4395264 | 22.91 | 20.26 | 21.23 | 21.43 | 21.59 | 22.29 | 22.31 | 21.21 | 21.24 |
| hsa-miR-924-4395265 | 40 | 40 | 40 | 40 | 40 | 40 | 40 | 35.72 | 40 |
| hsa-miR-92a-1*-4395248 | 28.13 | 27.42 | 28.84 | 28.58 | 28.62 | 27.81 | 30.36 | 30.06 | 29.72 |
| hsa-miR-92a-4395169 | 26.19 | 26.81 | 26.6 | 26.97 | 26.45 | 27.12 | 30.42 | 30.87 | 30.66 |
| hsa-miR-92b*-4395454 | 25.46 | 27.35 | 27.19 | 27.03 | 27.06 | 28.61 | 29.25 | 30.19 | 29.24 |
| hsa-miR-93*-4395250 | 26.11 | 27.2 | 26.96 | 27.05 | 26.71 | 27.1 | 30.95 | 30.27 | 30.08 |
| hsa-miR-933-4395287 | 40 | 31.05 | 40 | 31.72 | 40 | 40 | 40 | 40 | 40 |
| hsa-miR-93-4373302 | 23.69 | 24.65 | 24.42 | 24.59 | 24.12 | 24.64 | 27.76 | 28.17 | 27.97 |
| hsa-miR-934-4395288 | 40 | 40 | 40 | 40 | 40 | 34.97 | 40 | 40 | 40 |
| hsa-miR-935-4395289 | 30.01 | 29.69 | 29.35 | 29.49 | 29.52 | 29.46 | 30 | 29.64 | 28.98 |
| hsa-miR-936-4395290 | 40 | 35.86 | 40 | 40 | 40 | 40 | 40 | 40 | 40 |
| hsa-miR-937-4395291 | 31.3 | 32.03 | 40 | 40 | 31.43 | 33.98 | 40 | 40 | 40 |
| hsa-miR-938-4395292 | 31.23 | 31.4 | 32.65 | 32.53 | 32.78 | 31.64 | 35.7 | 40 | 40 |
| hsa-miR-939-4395293 | 25.61 | 25.3 | 25.85 | 25.64 | 25.95 | 25.74 | 25.43 | 25.73 | 25.3 |
| hsa-miR-941-4395294 | 28.53 | 29.03 | 28.94 | 29.6 | 28.41 | 29.09 | 30.93 | 31.74 | 30.37 |
| hsa-miR-942-4395298 | 32.18 | 33.55 | 32.97 | 33.02 | 33.46 | 34.57 | 36 | 35.38 | 40 |
| hsa-miR-943-4395299 | 40 | 35.2 | 33.22 | 34.07 | 34.3 | 31.58 | 36.61 | 40 | 31.01 |
| hsa-miR-9-4373285 | 18.7 | 19.1 | 18.68 | 18.91 | 18.7 | 18.77 | 20.06 | 20.97 | 20.63 |
| hsa-miR-944-4395300 | 40 | 40 | 35.63 | 40 | 40 | 40 | 40 | 40 | 40 |
| hsa-miR-95-4373011 | 29.4 | 28.65 | 28.19 | 27.9 | 27.84 | 28.51 | 27.65 | 28.08 | 27.96 |
| hsa-miR-96*-4395251 | 40 | 38 | 40 | 40 | 40 | 40 | 40 | 40 | 40 |
| hsa-miR-96-4373372 | 35.97 | 40 | 40 | 40 | 35.98 | 40 | 40 | 40 | 40 |
| hsa-miR-98-4373009 | 30.95 | 31.73 | 32.02 | 31.84 | 30.98 | 31.08 | 31.38 | 31.65 | 31.34 |
| hsa-miR-99a*-4395252 | 29.63 | 28.73 | 28.86 | 28.62 | 28.4 | 28.57 | 31.71 | 31.37 | 31.36 |
| hsa-miR-99a-4373008 | 26.23 | 25.3 | 25.34 | 25.15 | 24.62 | 24.63 | 26.72 | 26.96 | 26.51 |
| hsa-miR-99b*-4395307 | 30.09 | 32.53 | 31.79 | 30.4 | 30.87 | 32.99 | 32.39 | 32.05 | 32.3 |
| hsa-miR-99b-4373007 | 25.92 | 26.93 | 26.72 | 26.88 | 26.41 | 26.77 | 27.97 | 27.87 | 27.32 |
| MammU6-4395470 | 19.3 | 19.51 | 18.88 | 18.62 | 18.27 | 18.96 | 20.62 | 20.8 | 20.06 |
| MammU6-4395470 | 18.49 | 19.52 | 18.78 | 18.53 | 18.33 | 18.85 | 20.86 | 20.85 | 20.2 |
| RNU24-4373379 | 26.7 | 26.74 | 26.74 | 26.71 | 26.57 | 26.64 | 28.77 | 29.06 | 28.89 |
| RNU43-4373375 | 24.35 | 24.52 | 24.66 | 24.61 | 24.51 | 24.66 | 26.84 | 26.63 | 26.11 |
| RNU44-4373384 | 27.01 | 26.81 | 26.18 | 25.96 | 25.76 | 25.81 | 27.45 | 27.38 | 27.29 |
| RNU44-4373384 | 25.77 | 25.93 | 25.56 | 25.35 | 25.03 | 25.27 | 27.18 | 27.18 | 26.8 |
| RNU48-4373383 | 24.01 | 23.96 | 23.55 | 23.36 | 23.03 | 22.86 | 24.02 | 24.68 | 24.54 |
| RNU48-4373383 | 23.92 | 23.79 | 23.52 | 23.31 | 23.33 | 23.12 | 24.44 | 24.7 | 24.64 |
| RNU6B-4373381 | 25.89 | 26.75 | 26.86 | 26.85 | 26.7 | 27.07 | 28.76 | 28.61 | 28.05 |

**Figure S2. Quantile-Quantile plot of fold change differences in pre- and post-natal timepoints with week 14 as reference**

**Table S2. MicroRNAs with >2 SD fold change from 14 weeks timepoint in fetal only analysis**

| **detector** | **week14** | **week16** | **week17** | **week18** | **week19** | **week20** |
| --- | --- | --- | --- | --- | --- | --- |
| hsa-miR-923-4395264 | 24065.13 | 186139.35 | 76295.56 | 59686.85 | 46383.95 | 33298.68 |
| hsa-miR-128-4395327 | 550.52 | 1853.48 | 1447.46 | 1635.06 | 1860.32 | 2309.20 |
| hsa-miR-138-4395395 | 295.02 | 1071.95 | 1037.80 | 1716.35 | 1439.49 | 1610.37 |
| hsa-miR-204-4373094 | 573.90 | 1765.70 | 2032.88 | 1612.55 | 2859.09 | 2509.49 |
| hsa-miR-137-4373301 | 869.87 | 2639.45 | 2874.93 | 2967.69 | 3495.63 | 4279.35 |
| hsa-miR-567-4380944 | 569.94 | 823.73 | 501.22 | 163.72 | 72.07 | 294.72 |
| hsa-miR-501-5p-4373226 | 663.82 | 70.82 | 250.61 | 135.78 | 64.50 | 80.07 |
| hsa-miR-532-5p-4380928 | 882.01 | 438.38 | 354.42 | 355.85 | 347.61 | 170.45 |
| hsa-miR-887-4395485 | 531.77 | 159.35 | 278.07 | 169.50 | 184.99 | 69.22 |
| hsa-miR-449b-4381011 | 45.40 | 258.86 | 675.27 | 578.08 | 352.47 | 63.26 |
| hsa-miR-449a-4373207 | 19.76 | 517.72 | 1662.70 | 1132.37 | 465.08 | 0.00 |
| hsa-miR-25-4373071 | 919.47 | 447.59 | 364.38 | 309.79 | 340.46 | 201.30 |
| hsa-miR-216b-4395437 | 1291.34 | 123.30 | 65.31 | 198.79 | 220.00 | 322.50 |
| hsa-miR-122-4395356 | 47.00 | 566.54 | 0.00 | 25.90 | 81.65 | 0.00 |

*Numbers are unit less and represent relative expression

**Table S3. MicroRNAs with >2 SD fold change from 5-98 day timepoint in postnatal only analysis**

| **detector** | **D5_98** | **D443_502** | **D1630_1733** |
| --- | --- | --- | --- |
| hsa-miR-31-4395390 | 2748.43 | 12084.54 | 11986.08 |
| hsa-miR-885-3p-4395483 | 1273.33 | 1952.19 | 9021.00 |
| hsa-miR-34a-4395168 | 1171.70 | 4740.67 | 5591.70 |
| hsa-miR-19a-4373099 | 10473.02 | 3877.40 | 2519.76 |
| hsa-miR-431-4395173 | 783.83 | 3398.95 | 1819.18 |
| hsa-miR-565-4380942 | 262.18 | 1105.80 | 1572.75 |
| hsa-miR-29b-4373288 | 119.79 | 1105.80 | 770.19 |
| hsa-miR-23a-4373074 | 2279.32 | 942.84 | 634.32 |
| hsa-miR-499-5p-4381047 | 938.62 | 267.03 | 608.48 |
| hsa-miR-135a-4373140 | 1727.40 | 600.86 | 559.92 |
| hsa-miR-508-3p-4373233 | 1108.50 | 319.77 | 164.17 |
| hsa-miR-135b-4395372 | 2546.66 | 424.87 | 129.70 |
| hsa-miR-542-5p-4395351 | 687.11 | 108.45 | 29.43 |
| hsa-miR-505-4395200 | 550.42 | 501.77 | 0.00 |
| hsa-miR-662-4381010 | 520.73 | 0.00 | 0.00 |

*Numbers are unit less and represent relative expression

**Table S4. MicroRNAs with >2 SD fold change from 14 week timepoint in combined fetal and postnatal analysis**

| **detector** | **week14** | **week16** | **week17** | **week18** | **week19** | **week20** | **D5_98** | **D443_502** | **D1630_1733** |
| --- | --- | --- | --- | --- | --- | --- | --- | --- | --- |
| hsa-miR-132-4373143 | 1612.02 | 2549.54 | 2400.82 | 2619.59 | 2227.70 | 2357.72 | 53394.00 | 62904.46 | 71669.51 |
| hsa-miR-22-4373079 | 888.15 | 1035.44 | 935.32 | 1196.93 | 700.06 | 663.14 | 14012.14 | 15617.49 | 17307.04 |
| hsa-miR-31-4395390 | 188.01 | 188.19 | 87.99 | 231.54 | 89.35 | 104.92 | 2748.43 | 12084.54 | 11986.08 |
| hsa-miR-885-3p-4395483 | 220.50 | 0.00 | 184.73 | 0.00 | 354.92 | 0.00 | 1273.33 | 1952.19 | 9021.00 |
| hsa-miR-139-3p-4395424 | 425.98 | 429.35 | 458.03 | 645.88 | 498.46 | 631.74 | 4527.16 | 6658.01 | 8593.75 |
| hsa-miR-17-4395419 | 81508.14 | 72516.59 | 71186.27 | 59686.85 | 63362.34 | 52251.24 | 17859.33 | 11835.84 | 7481.29 |
| hsa-miR-138-4395395 | 295.02 | 1071.95 | 1037.80 | 1716.35 | 1439.49 | 1610.37 | 7561.15 | 9613.69 | 7429.62 |
| hsa-miR-146b-5p-4373178 | 133.87 | 225.35 | 171.17 | 190.70 | 227.75 | 282.71 | 2186.47 | 6520.99 | 6649.69 |
| hsa-miR-106a-4395280 | 63508.25 | 55724.48 | 51393.97 | 44611.42 | 45745.37 | 41856.89 | 13723.78 | 9883.97 | 6558.14 |
| hsa-miR-34a-4395168 | 89.55 | 112.67 | 67.15 | 82.43 | 99.14 | 44.11 | 1171.70 | 4740.67 | 5591.70 |
| hsa-miR-223-4395406 | 182.87 | 303.60 | 219.69 | 394.84 | 359.87 | 378.25 | 3601.52 | 9680.56 | 4605.28 |
| hsa-miR-20a-4373286 | 43377.42 | 41649.85 | 35593.14 | 25445.54 | 33720.49 | 25945.15 | 8565.90 | 4806.85 | 3167.37 |
| hsa-miR-383-4373018 | 21.78 | 113.46 | 146.96 | 153.82 | 148.19 | 114.02 | 2691.86 | 2858.17 | 2590.60 |
| hsa-miR-146a-4373132 | 132.02 | 303.60 | 308.54 | 384.04 | 278.46 | 254.79 | 3245.87 | 3215.61 | 2572.71 |
| hsa-miR-19a-4373099 | 54525.99 | 57291.10 | 47952.27 | 33809.13 | 32123.42 | 24376.06 | 10473.02 | 3877.40 | 2519.76 |
| hsa-miR-539-4378103 | 92.07 | 152.86 | 125.31 | 148.58 | 137.31 | 68.74 | 701.54 | 1796.38 | 2334.78 |
| hsa-miR-219-5p-4373080 | 61.17 | 59.55 | 10.92 | 10.67 | 8.58 | 19.34 | 598.16 | 1808.87 | 2133.60 |
| hsa-miR-7-4378130 | 38.98 | 143.61 | 281.95 | 198.79 | 55.38 | 47.94 | 1412.85 | 3693.76 | 1909.62 |
| hsa-miR-433-4373205 | 112.57 | 201.69 | 156.42 | 244.74 | 72.57 | 78.97 | 1374.21 | 2858.17 | 1844.57 |
| hsa-miR-485-5p-4373212 | 350.84 | 460.17 | 47.15 | 459.88 | 0.00 | 0.00 | 1943.43 | 2049.24 | 1769.43 |
| hsa-miR-150-4373127 | 100.75 | 112.67 | 140.00 | 217.54 | 159.93 | 144.33 | 1472.85 | 1783.97 | 1721.05 |
| hsa-miR-497-4373222 | 46.03 | 258.86 | 97.63 | 158.15 | 196.90 | 180.17 | 1535.39 | 1136.89 | 1616.96 |
| hsa-miR-668-4395181 | 141.50 | 107.34 | 67.62 | 157.05 | 39.71 | 16.49 | 641.09 | 1641.59 | 1498.26 |
| hsa-miR-187-4373307 | 36.88 | 108.84 | 43.69 | 138.63 | 145.14 | 17.43 | 1691.85 | 2420.14 | 1081.69 |
| hsa-miR-770-5p-4395189 | 45.09 | 154.99 | 85.00 | 133.91 | 77.24 | 97.90 | 506.49 | 1235.50 | 961.45 |
| hsa-miR-490-3p-4373215 | 43.85 | 238.20 | 127.94 | 161.47 | 89.35 | 101.35 | 1483.09 | 949.40 | 961.45 |
| hsa-miR-212-4373087 | 29.54 | 55.95 | 45.86 | 58.69 | 34.57 | 25.51 | 857.74 | 936.33 | 890.87 |
| hsa-miR-517a-4395513 | 38.98 | 81.35 | 6.49 | 14.88 | 57.73 | 35.34 | 506.49 | 962.65 | 825.47 |
| hsa-miR-338-3p-4395363 | 32.33 | 43.59 | 45.23 | 39.26 | 70.10 | 30.55 | 358.14 | 798.35 | 819.77 |
| hsa-miR-29b-4373288 | 38.18 | 25.04 | 11.00 | 0.00 | 5.21 | 10.01 | 119.79 | 1105.80 | 770.19 |
| hsa-miR-567-4380944 | 569.94 | 823.73 | 501.22 | 163.72 | 72.07 | 294.72 | 1131.79 | 695.00 | 708.72 |
| hsa-miR-409-5p-4395442 | 228.28 | 101.55 | 0.00 | 111.83 | 95.76 | 41.73 | 325.02 | 427.82 | 679.85 |
| hsa-miR-654-5p-4381014 | 279.10 | 211.72 | 245.45 | 258.70 | 141.17 | 22.52 | 594.03 | 1315.03 | 665.86 |
| hsa-miR-499-5p-4381047 | 16.62 | 0.00 | 0.00 | 22.24 | 184.99 | 0.00 | 938.62 | 267.03 | 608.48 |
| hsa-miR-127-5p-4395340 | 10.74 | 77.49 | 60.52 | 8.55 | 51.67 | 62.82 | 303.26 | 142.11 | 567.73 |
| hsa-miR-501-5p-4373226 | 663.82 | 70.82 | 250.61 | 135.78 | 64.50 | 80.07 | 66.46 | 184.94 | 439.30 |
| hsa-miR-518d-3p-4373248 | 43.25 | 0.00 | 221.22 | 58.29 | 65.41 | 183.95 | 711.34 | 855.65 | 385.10 |
| hsa-miR-130b-4373144 | 3528.05 | 1853.48 | 1467.67 | 1546.86 | 1380.85 | 1281.11 | 280.99 | 302.52 | 332.93 |
| hsa-miR-887-4395485 | 531.77 | 159.35 | 278.07 | 169.50 | 184.99 | 69.22 | 0.00 | 750.07 | 312.79 |
| hsa-miR-15b-4373122 | 2865.67 | 1853.48 | 1685.91 | 1291.76 | 1653.54 | 924.92 | 510.01 | 375.03 | 310.63 |
| hsa-miR-142-3p-4373136 | 45.09 | 45.13 | 49.16 | 48.68 | 26.56 | 35.83 | 462.85 | 809.49 | 302.14 |
| hsa-miR-92a-4395169 | 2477.48 | 1986.51 | 1844.88 | 1282.84 | 1597.21 | 1170.72 | 397.38 | 319.77 | 285.84 |
| hsa-miR-451-4373360 | 26.26 | 38.21 | 22.62 | 27.76 | 22.81 | 25.16 | 546.62 | 478.00 | 233.79 |
| hsa-miR-449a-4373207 | 19.76 | 517.72 | 1662.70 | 1132.37 | 465.08 | 0.00 | 242.93 | 427.82 | 164.17 |
| hsa-miR-508-3p-4373233 | 44.78 | 96.07 | 100.38 | 66.03 | 77.78 | 56.23 | 1108.50 | 319.77 | 164.17 |
| hsa-miR-135b-4395372 | 11622.69 | 11796.11 | 9471.07 | 10121.43 | 9887.14 | 9301.05 | 2546.66 | 424.87 | 129.70 |
| hsa-miR-18a-4395533 | 906.81 | 582.46 | 454.87 | 334.33 | 377.76 | 343.26 | 137.61 | 114.63 | 84.39 |
| hsa-miR-25-4373071 | 919.47 | 447.59 | 364.38 | 309.79 | 340.46 | 201.30 | 79.58 | 106.96 | 66.21 |
| hsa-miR-18b-4395328 | 585.96 | 417.61 | 227.44 | 159.25 | 340.46 | 350.48 | 147.48 | 150.21 | 59.26 |
| hsa-miR-301b-4395503 | 783.97 | 341.57 | 356.88 | 225.21 | 313.29 | 261.96 | 74.25 | 54.22 | 45.23 |
| hsa-miR-542-5p-4395351 | 85.31 | 0.00 | 22.31 | 84.75 | 44.06 | 0.00 | 687.11 | 108.45 | 29.43 |
| hsa-miR-33b-4395196 | 417.22 | 0.00 | 173.56 | 496.32 | 150.26 | 166.94 | 0.00 | 4675.40 | 0.00 |
| hsa-miR-505-4395200 | 602.43 | 246.60 | 218.17 | 309.79 | 326.59 | 462.46 | 550.42 | 501.77 | 0.00 |
| hsa-miR-216b-4395437 | 1291.34 | 123.30 | 65.31 | 198.79 | 220.00 | 322.50 | 0.00 | 107.70 | 0.00 |
| hsa-miR-662-4381010 | 147.51 | 198.92 | 0.00 | 94.69 | 20.55 | 85.22 | 520.73 | 0.00 | 0.00 |
| hsa-miR-122-4395356 | 47.00 | 566.54 | 0.00 | 25.90 | 81.65 | 0.00 | 135.71 | 0.00 | 0.00 |

*Numbers are unit less and represent relative expression
